# Inpp5e is crucial for photoreceptor outer segment maintenance

**DOI:** 10.1242/jcs.263814

**Published:** 2025-02-21

**Authors:** Mohona Gupta, Tylor R. Lewis, Michael W. Stuck, William J. Spencer, Natalia V. Klementieva, Vadim Y. Arshavsky, Gregory J. Pazour

**Affiliations:** ^1^ Program in Molecular Medicine, University of Massachusetts Chan Medical School, Suite 213 Biotech II, 373 Plantation Street, Worcester, MA 01605, USA; ^2^ Morningside Graduate School of Biological Sciences, University of Massachusetts Chan Medical School, 55 Lake Avenue North, Worcester, MA 01655, USA; ^3^ Department of Ophthalmology, Duke University School of Medicine, 2351 Erwin Rd, Durham North Carolina, NC 27710, USA; ^4^ Ophthalmology and Visual Sciences, SUNY Upstate Medical University, 505 Irving Avenue, Syracuse, NY 13210, USA

**Keywords:** Inpp5e, Retinal degeneration, Cilia, Vision

## Abstract

In humans, inositol polyphosphate-5-phosphatase E (INPP5E) mutations cause retinal degeneration as part of Joubert and MORM syndromes and can also cause non-syndromic blindness. In mice, mutations cause a spectrum of brain, kidney and other anomalies and prevent the formation of photoreceptor outer segments. To further explore the function of Inpp5e in photoreceptors, we generated conditional and inducible knockouts of mouse *Inpp5e* where the gene was deleted either during outer segment formation or after outer segments were fully formed. In both cases, the loss of Inpp5e led to severe defects in photoreceptor outer segment morphology and ultimately photoreceptor cell loss. The primary morphological defect consisted of outer segment shortening and reduction in the number of newly forming discs at the outer segment base. This was accompanied by structural abnormalities of the Golgi, mislocalized rhodopsin and an accumulation of extracellular vesicles. In addition, knockout cells showed disruption of the actin network. Together, these data demonstrate that Inpp5e plays a crucial role in maintaining the outer segment and the normal process of outer segment renewal depends on the activity of this enzyme.

## Teaser

**Summary:** Loss of the inositol phosphatase INPP5E from mature photoreceptors leads to rapid outer segment degeneration marked by a failure to form new discs and structural abnormalities of the Golgi complex.

## INTRODUCTION

Primary cilia are evolutionarily conserved microtubule-based organelles that regulate cellular signaling pathways and maintain cellular homeostasis. Defects in cilia cause a large group of diseases known as ciliopathies. The ciliopathies include blindness as the light-sensitive outer segment of rod and cone photoreceptors develops from a primary cilium. To form the outer segment, proteins and lipids synthesized in the inner segment of the cell body are transported into the cilium and the ciliary membrane is remodeled into a stack of flattened discs enriched in visual pigments and signaling molecules. In mice, photoreceptor cells become post mitotic at around post-natal day (P)3 and the process of forming an outer segment from the primary cilium begins at around P9 or P10 with outer segments reaching full length at around P25 (LaVail, 1973). Disc formation is driven by the actin cytoskeleton where F-actin polymerization pushes the ciliary membrane out to form a flattened ciliary membrane evagination. As the discs form, the actin filaments depolymerize and, in rods, the flattened membrane protrusions are further remodeled to form enclosed discs that are no longer continuous with the ciliary membrane (Spencer et al., 2020). The process of disc formation persists throughout an life of an individual as ∼10% of the discs are shed from the distal tip daily and are replenished by new discs forming at the base (LaVail, 1973; Young, 1967). Mutations affecting the development or maintenance of the outer segment lead to congenital or degenerative diseases of the retina (Gupta and Pazour, 2023).

Pathogenic variants in *INPP5E* lead to retinal degeneration as part of MORM (Jacoby et al., 2009) and Joubert syndromes (Bielas et al., 2009), and as non-syndromic isolated retinal degeneration (Sangermano et al., 2021). The variants causing MORM syndrome typically affect the CAAX box at the C-terminal end of the protein. This motif is prenylated, causing the protein to be peripherally associated with membrane. MORM syndrome impairs INPP5E ciliary localization and is characterized by impaired intellectual development, truncal obesity, micropenis and retinal dystrophy (Hampshire et al., 2006). Joubert syndrome variants typically affect the enzymatic activity of the protein and yield hypoplasia of the cerebellar vermis with variable presentations of retinal dystrophy and renal anomalies (Bielas et al., 2009; Travaglini et al., 2013; Van De Weghe et al., 2022). The variants associated with isolated retinal degeneration are found outside of the catalytic domain or are subtle substitutions, such as Asp to Glu or Arg to His (Sangermano et al., 2021).

Inpp5e was originally identified as a Golgi-enriched phosphoinositide phosphatase and proposed to regulate Golgi transport (Kong et al., 2000). It was later found to regulate autophagosome fusion with lysosomes (Hasegawa et al., 2016). Inpp5e activity converts the phosphoinositides PI(3,5)P_2_, PI(4,5)P_2_, and PI(3,4,5)P_3_ into PI(3)P, PI(4)P and PI(3,4)P_2_, respectively (Kisseleva et al., 2000; Kong et al., 2000). Recently Inpp5e has emerged as an important regulator of ciliary phosphoinositide composition where it functions to convert PI(4,5)P_2_ into PI(4)P in the ciliary shaft and PI(3,4,5)P_3_ into PI(3,4)P_2_ in the transition zone (Dyson et al., 2017). Under normal conditions, the plasma membrane and ciliary membrane near the ciliary base are enriched in PI(4,5)P_2_, whereas the more distal ciliary membrane has low PI(4,5)P_2_ but elevated PI(4)P due to the action of Inpp5e. This is crucially important for the ability of the cilium to regulate Hedgehog signaling (Chávez et al., 2015; Constable et al., 2020; Garcia-Gonzalo et al., 2015) and also regulates removal of cilia through an actin-mediated decapitation of the ciliary tip (Phua et al., 2017). At the transition zone, the lack of Inpp5e activity disrupts the localization of numerous transition zone proteins suggesting that assembly of this structure is dependent on the ratio of PI(3,4,5)P_3_ to PI(3,4)P_2_ (Dyson et al., 2017). The cellular localization of Inpp5e might be regulated, as the loss of the small GTPase Arl16 redistributes Inpp5e from the cilium to the Golgi complex (Dewees et al., 2022).

Germline deletion of *Inpp5e* in mouse causes brain malformations (anencephaly and exencephaly), polydactyly and other skeletal anomalies, cystic kidneys and bilateral anophthalmia (Jacoby et al., 2009). Deletion of *Inpp5e* in the retina with *Six3-Cre* blocked the formation of discs from the ciliary membrane and caused rapid retinal degeneration (Sharif et al., 2021). This Cre is expressed embryonically in the retina prior to cells becoming post-mitotic and forming primary cilia (Furuta et al., 2000) indicating that Inpp5e is crucial to early stages of photoreceptor outer segment development. To overcome challenges posed by embryonic lethality and to uncover Inpp5e function in later stages of outer segment formation and in photoreceptor maintenance, we utilized two distinct conditional mutant mouse models. In the first model, an *Inpp5e* floxed allele was deleted by rod-specific *iCre75* during photoreceptor outer segment maturation. In the second model, the *Inpp5e* floxed allele was deleted by the tamoxifen inducible rod-specific *Pde6g^CreERT2^*, which allowed us to analyze Inpp5e function in mature photoreceptors after outer segment morphogenesis was complete. In both cases, the loss of Inpp5e led to rapid photoreceptor degeneration. The degeneration was accompanied by shortening of the outer segments, malformations of the Golgi, accumulation of rhodopsin in the cell body, abnormal actin cytoskeleton, and the presence of abundant extracellular vesicles around photoreceptor somas. These findings suggest Inpp5e plays a crucial role in outer segment maintenance.

## RESULTS

### Rod-specific Inpp5e knockout leads to rapid vision loss

To elucidate the role of Inpp5e in photoreceptors, we deleted a floxed allele of *Inpp5e* (Dyson et al., 2017) using two Cre recombinase lines. The first, *iCre75*, utilizes a rod opsin promoter to selectively express Cre recombinase in rod photoreceptor cells. In this line, Cre expression begins at around postnatal day 7 (P7) (Li et al., 2005). Outer segment elongation starts at around P9 or P10 (LaVail, 1973; Obata and Usukura, 1992; Pearring et al., 2013), thus it is expected that pathology would start during the elongation phase due to the time required for the gene to be deleted and the gene products decay. The second, *Pde6g^CreERT2^* expresses a tamoxifen-inducible Cre recombinase under the control of the endogenous rod-specific *Pde6g* promoter allowing for *Inpp5e* ablation in mature rods. The *Inpp5e* floxed allele contains loxP sites flanking the catalytic domain and is expected to produce a null allele after deletion (Dyson et al., 2017).

**Fig. 1.**
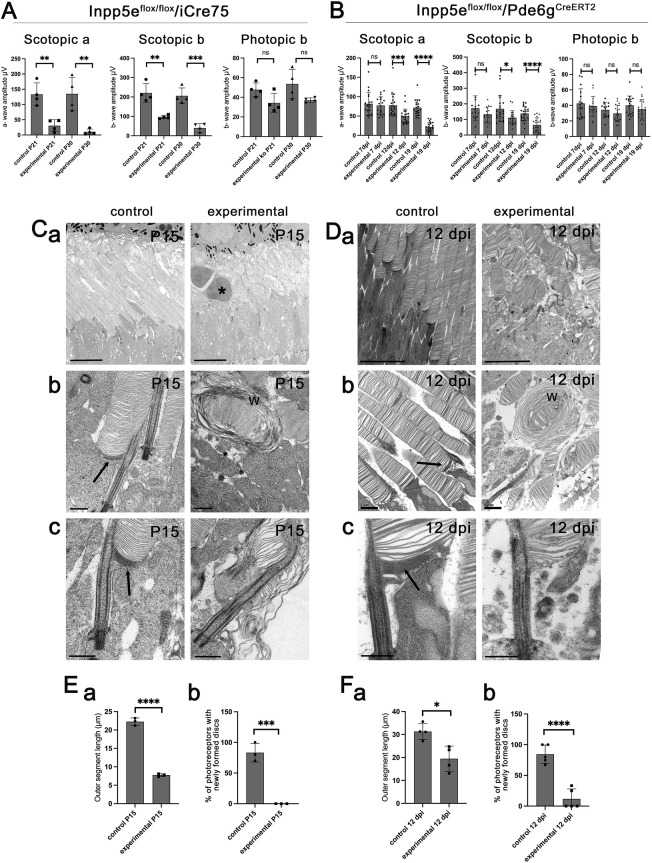
**Loss of Inpp5e leads to rapid vision loss.** (A) ERGs of *Inpp5e^flox/flox^* (control) and *Inpp5e^flox/flox^/iCre75* (experimental) littermates examined at P21 and P30. Each point represents the mean response from one animal. (B) ERGs of *Inpp5e^flox/flox^/Pde6g^CreERT2^* littermates treated with vehicle (control) or tamoxifen (experimental) and examined at 7, 12 and 19 dpi. Each point represents the mean response from one animal. (C) TEM images of *Inpp5e^flox/flox^* (control) and *Inpp5e^flox/flox^*/*iCre75* (experimental) littermates examined at P15. Scale bars: 5 µm (Ca), 0.5 µm (Cb,Cc). The asterisk marks the nuclei of a dead photoreceptor, w marks whorls of membrane, arrows point to darkened membrane at the base of outer segments. (D) TEM images of *Inpp5e^flox/flox^/Pde6g^CreERT2^* littermates treated with vehicle (control) or tamoxifen (experimental) examined 12 dpi. Scale bars: 5 µm (Da), 0.5 µm (Db,Dc). w marks membrane whorls, arrows point to darkened membrane at the base of outer segments. (Ea,Fa) Average outer segment length as measured in TEM of *iCre75* and *Pde6g^CreERT2^* animals as P15 and 12 dpi, respectively. Each point is the average from one animal. (Eb,Fb) Percentage of photoreceptors with newly formed discs in TEMs of *iCre75* and *Pde6g^CreERT2^* animals as P15 and 12 dpi, respectively. Each point is the mean from one animal. In all panels, error bars represent s.d. *P<0.05; **P<0.01; ***P<0.001; ****P<0.0001; ns, not significant (unpaired two-tailed *t*-test).

Electroretinography (ERG) was used to gauge photoreceptor function. In the *iCre75* line, experimental (*Inpp5e^flox/flox^*/*iCre75*) animals showed a clear reduction in retinal function at P21 (Fig. 1A). The retinal dysfunction was evident in both the initial rod photoreceptor response to light (scotopic a wave) and the downstream responses (scotopic b wave) when compared to control (*Inpp5e^flox/+^*/*iCre75*) animals. Scotopic a and b waves were nearly absent at P30, although a residual signal was observed that could be driven by cells where the Cre was not effective or in cells where the outer segments have not yet fully degenerated. Cone-driven responses (photopic b wave) were not significantly affected at these points. This is expected, as we deleted the *Inpp5e* gene in rod photoreceptors and the time points examined were too early for secondary cone loss.

For analysis of Inpp5e in mature photoreceptors, *Inpp5e^flox/flox^/Pde6g^CreERT2^* mice were treated with tamoxifen (experimental) or vehicle (control). Initially, we examined retinas at 30 days post last injection (dpi), but at this point the retinas were highly degenerated as observed by a severely thinned outer nuclear layer and little to no detectable outer segment layer (Fig. S1). To identify points early in degeneration where the effects on the retina are more likely directly caused by the loss of Inpp5e rather than the indirect effects of cell death, we carried out ERGs to monitor visual function at 7, 12 and 19 dpi (Fig. 1B). At 7 dpi, ERG responses of experimental mice were not different from those of controls, but by 12 dpi, scotopic a- and b-waves were reduced, and decreased further at 19 dpi. No significant difference in cone-driven responses (photopic b-waves) were observed at any time point, consistent with the *Inpp5e* knockout being rod specific.

### Loss of Inpp5e blocks new disc formation

Transmission electron microscopy (TEM) was used to assess photoreceptor structure. For *iCre75* deletion, dysfunction as measured by ERG was already evident at P21 (Fig. 1A), prompting tissue collection at P15 in order to survey the pathology earlier in disease progression. For *Pde6g^CreERT2^* animals, we collected tissue at 12 dpi, as this was the point when dysfunction was first detected by ERG. The pathology detected by TEM was similar in retinas collected from both lines (Fig. 1C,D). Both models, most experimental outer segments were irregularly shaped and shortened to about half as long as the control outer segments (Fig. 1Ca,Da,Ea,Fa). Structural abnormalities included malformed discs and occasional membrane whorls (Fig. 1Cb,Db). In the *iCre75* model, we observed occasional nuclei in the outer segment layer suggesting photoreceptor cell death was occurring (Fig. 1Ca). To examine the formation of new discs, we contrasted tissue with tannic acid and uranyl acetate. Owing to its low membrane permeability, tannic acid labels cell surface-exposed membranes more strongly than internal membranes. Hence, this technique labels the membranes of newly forming discs exposed to the extracellular environment more darkly than membranes of fully formed discs enclosed inside the outer segment (Ding et al., 2015) (Fig. 1Cc,Dc). In both lines, darkly stained discs at the base of the outer segment were observed in ∼75% of cells in control animals but they were almost never seen in experimental animals (Fig. 1Eb,Fb). This observation suggests that new disc formation does not occur when Inpp5e is missing.

### Inpp5e knockouts mislocalize rhodopsin

**Fig. 2.**
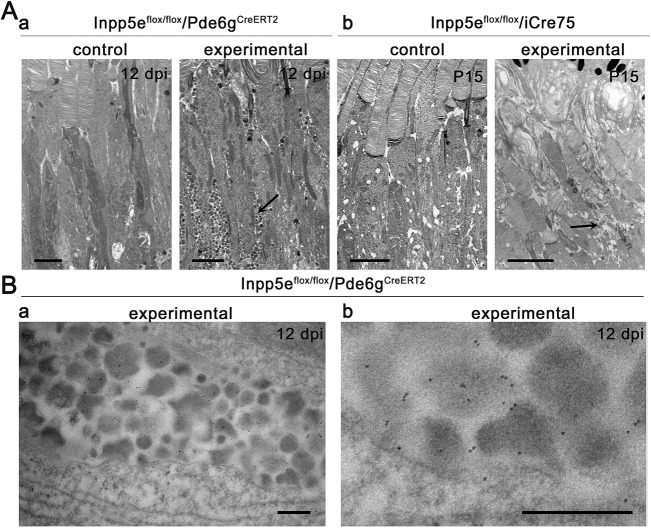
**Loss of Inpp5e causes loss of outer segment integrity and extracellular vesicle accumulation.** (Aa) TEM of *Inpp5e^flox/flox^/Pde6g^CreERT2^* littermates treated with vehicle (control) or tamoxifen (experimental) examined 12 days dpi. (Ab) TEM of *Inpp5e^flox/flox^/iCre75* littermates examined at P15. Images of inner segments reveal accumulation of vesicles (arrow) within the inner segment region of photoreceptors in the experimental group, which is absent in the control group. Scale bars: 2 µm. (B) Immunogold EM shows rhodopsin localization in the extracellular vesicles in the experimental group. Scale bars: 300 nm. Images are representative of eyes collected from four animals (A) and two animals (B).

In addition to the defects observed in the outer segments, the inner segment region of experimental animals from both lines contained large amounts of extracellular vesicles (Figs 1Ca,Da and 2A) similar to those in models with rhodopsin mislocalization (reviewed in Spencer, 2023). To investigate whether rhodopsin mistrafficking contributes to the formation of extracellular vesicles when Inpp5e is missing, we conducted rhodopsin immunogold electron microscopy. The vesicles labeled strongly with anti-rhodopsin antibodies, consistent with them containing rhodopsin (Fig. 2B).

**Fig. 3.**
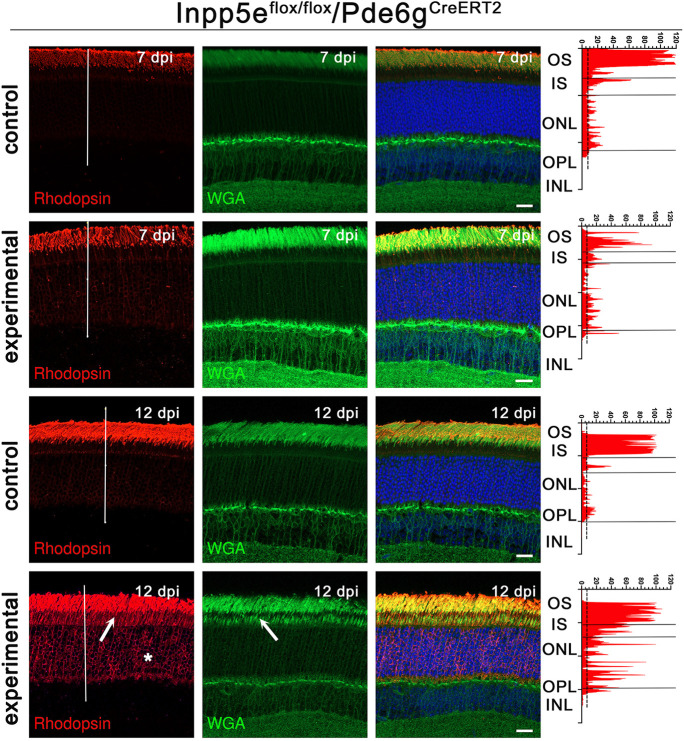
**Loss of Inpp5e causes rhodopsin mislocalization.** Confocal images of retinal sections (agarose embedded) of *Inpp5e^flox/flox^/Pde6g^CreERT2^* littermates treated with vehicle (control) or tamoxifen (experimental) at 7 and 12 days dpi immunostained with anti-rhodopsin clone 4D2 (red) and WGA (green). Scale bars: 20 µm. Each image is a maximum intensity projection of 20 images taken at 0.7-µm intervals. Rhodopsin intensity along the white line is shown on the right side of the images. Arrows point to mislocalized rhodopsin or WGA in the inner segment. The asterisk points to rhodopsin in the nuclear layer. OS, outer segment; IS, inner segment; ONL, outer nuclear layer; OPL, outer plexiform layer; INL, inner nuclear layer. Images are representative of eyes collected from three animals.

Rhodopsin, the visual pigment in rod photoreceptors, is synthesized in the inner segment and transported to the outer segment where it is highly concentrated. Accordingly, wild-type mice typically display weak rhodopsin signal in the inner segments and strong signal in the outer segments. Retinal degeneration-causing mutations often misaccumulate rhodopsin in the inner segment, nuclear layer and synaptic regions. In the *Pde6g^CreERT2^* model, rhodopsin mislocalization was negligible at 7 dpi but became prominent by 12 dpi (Fig. 3). *Inpp5e/iCre75* experimental animals showed rhodopsin accumulation throughout the entire photoreceptor cell body as early as P15, which was further increased at P21 (Fig. S2).

### Golgi structure is disrupted by loss of Inpp5e

**Fig. 4.**
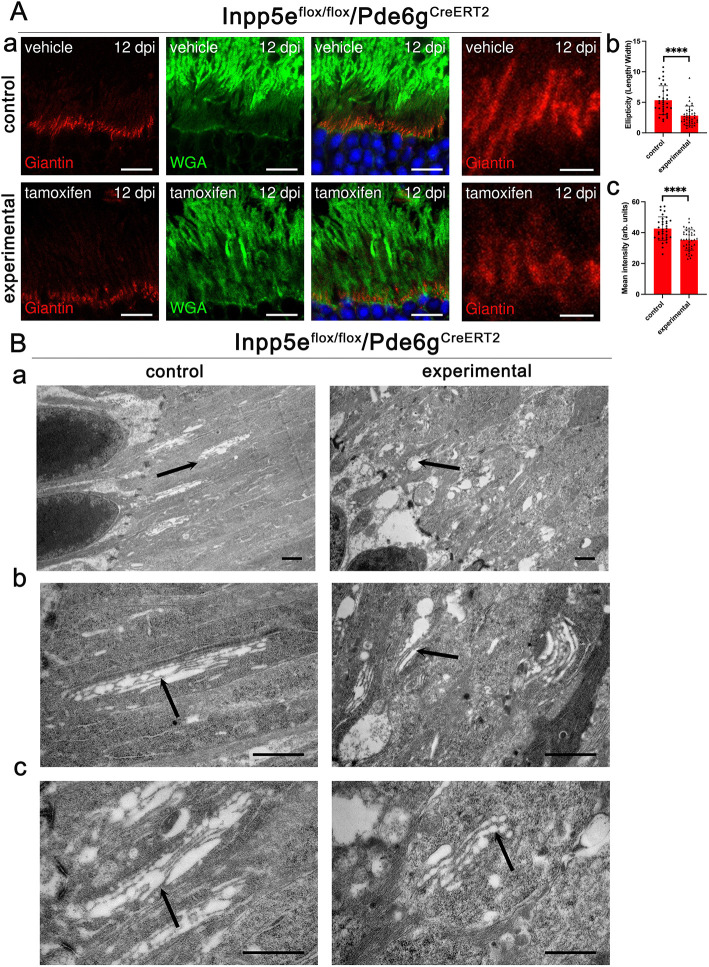
**Loss of Inpp5e affects Golgi structure.** (Aa) Confocal images of retinal sections (cryosections) of *Inpp5e^flox/flox^/Pde6g^CreERT2^* littermates treated with vehicle (control) or tamoxifen (experimental) at 12 dpi immunostained with anti-giantin (red) and WGA (green). Scale bars: 10 µm. Each image is a single plane of a *z*-stack. (Ab) Quantification of ellipticity. Each dot represents the length/width of a Golgi structure stained with giantin. (Ac) Intensity of giantin label per Golgi structure. Error bars represent s.d. ****P<0.0001 (unpaired two-tailed *t*-test). (B) TEM images showing the inner segment above the limiting membrane where Golgi structures are located. Arrows point to Golgi stacks. Scale bars: 1 µm. Images are representative of eyes collected from three animals (A) and four animals (B).

Wheat germ agglutinin (WGA) binds to glycosylated proteins, such as rhodopsin, and normally labels outer but not inner segments. In the *Pde6g^CreERT2^* animals, WGA staining was fairly normal at 7 dpi, but was significantly increased in the inner segments and the outer nuclear layer by 12 dpi (Fig. 3). In *iCre75* experimental animals, WGA staining was slightly elevated in the inner segment layer by P15 and became prominent by P21 (Fig. S2). WGA staining only partially overlapped with rhodopsin staining, indicating that the inner segment staining is not solely due to rhodopsin mislocalization. The strongest WGA staining in the inner segment was seen just above the nuclear layer where Golgi complexes are located. To determine whether the Golgi structure was affected by the loss of Inpp5e, we stained retinas with WGA and the cis-medial Golgi complex marker giantin (also known as GOLGB1) (Nozawa et al., 2002). In control animals, giantin prominently labeled elongated structures in the expected Golgi location (Fig. 4Aa). In 12 dpi experimental animals, the intensity of giantin label was slightly reduced and the structures observed were more rounded than in control (Fig. 4A). TEM of control retinas showed an elongated Golgi structure typical of rod cells, whereas the Golgi structures in experimental retinas were harder to identify and the ones observed were typically more swollen (Fig. 4B), consistent with light microscopy observations (Fig. 4A).

**Fig. 5.**
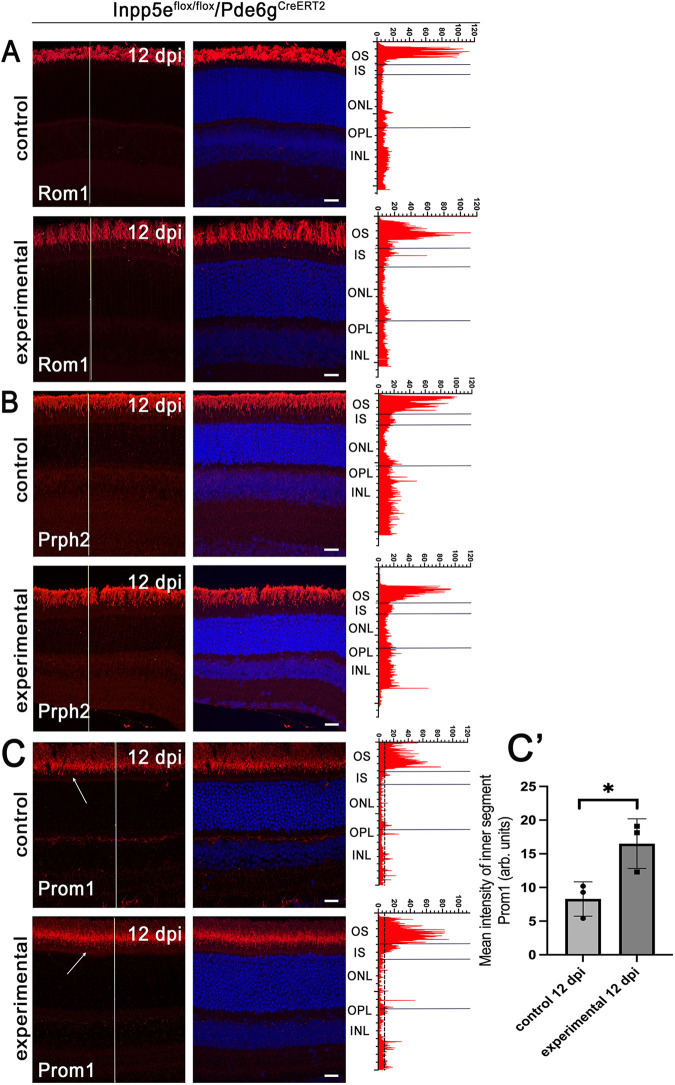
**Loss of Inpp5e alters distribution of Prom1.** (A–C) Confocal images of retinal sections (agarose embedded) of vehicle (control) and tamoxifen (experimental)-treated *Inpp5e^flox/flox^/Pde6g^CreERT2^* littermates at 12 dpi. Sections were stained with DAPI (blue) and for Rom1 (A) Prph2 (B), and Prom1 (C) in red. Note increased Prom1 in the inner segments (arrow) of the experimental animals compared to controls. Scale bars: 20 µm. Each image is a maximum intensity projection of 20 images taken at 0.7-µm intervals. The intensity of the red channel along the white line is shown on the right side of the images. OS, outer segment; IS, inner segment; ONL, outer nuclear layer; OPL, outer plexiform layer; INL, inner nuclear layer. (C′) Quantification of Prom1 fluorescence intensity in the inner segments of control and experimental retinas (*n*=3). Each point represents an individual measurement from one animal, with mean±s.d. shown. *P<0.05 (unpaired two-tailed *t*-test). Images in A and B are representative of eyes collected from three animals.

To analyze the extent of protein mislocalization from outer segments in the absence of Inpp5e, we surveyed the subcellular distribution of integral membrane proteins peripherin-2 (Prph2), retinal outer segment membrane protein 1 (Rom1), and prominin-1 (Prom1) (Fig. 5; Fig. S3). Prph2 and Rom1 support disc rim formation (Stuck et al., 2016), and are normally localized throughout the outer segment. Prom1 is important for disc morphogenesis and is normally found at the base of the outer segment (Yang et al., 2008). We did not observe any notable changes in the distribution of Prph2 or Rom1 in either *Inpp5e*-knockout model (Fig. 5A,B; Fig. S3A,B). In contrast, there was a minor inner segment accumulation of Prom1 (Fig. 5C; Fig. S3C). A possible explanation for the different effects of the Inpp5e loss on the distribution of these proteins might lie in their distinct trafficking pathways. Prph2 and Rom1 are delivered to the outer segment directly from the endoplasmic reticulum (Tian et al., 2014), whereas rhodopsin, and potentially, Prom1, are processed through the Golgi before reaching their final destination (Campos et al., 2011; Papermaster et al., 1986; Sung and Tai, 2000). This difference in trafficking routes could account for the accumulation of Prom1 in the inner segment of our *Inpp5e*-knockout models. Our findings are similar to those observed in Sharif et al. except that the deletion of *Inpp5e* by *Six3-Cre* did not yield a notable increase in inner segment Prom1 (Sharif et al., 2021).

We also analyzed the distribution of the SNARE protein syntaxin-3 (Stx3), which is normally excluded from the outer segment but present in the inner segment and synaptic terminal (Datta et al., 2015). We found no difference in Stx3 distribution between control and *Inpp5e*-knockout rods (Fig. S4). Stx3 accumulates in the outer segment in models of retinal degeneration caused by defects in the BBSome (Datta et al., 2015; Dilan et al., 2018; Hsu et al., 2017), but not in *Ift20* and *Ift172* knockouts (Lewis et al., 2024). The lack of Stx3 accumulation in *Inpp5e* knockouts could indicate that *Inpp5e* loss does not affect the trafficking of Stx3. Alternatively, the failure of new disc assembly could close the pathway for Stx3 entry into the cilium, thus indirectly preventing its accumulation.

### Loss of Inpp5e disrupts the actin cytoskeleton

**Fig. 6.**
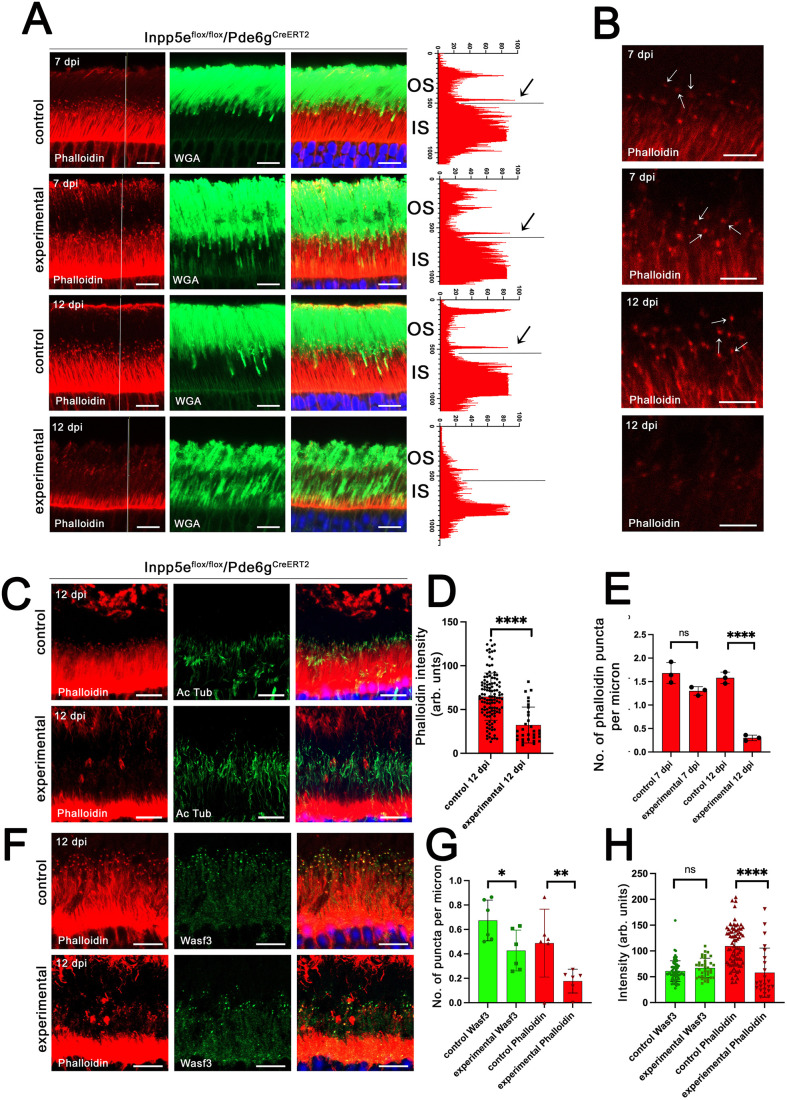
**Loss of Inpp5e disrupts the actin cytoskeleton.** (A) Confocal images of retinal sections of vehicle (control)- and tamoxifen (experimental)-treated *Inpp5e^flox/flox^/Pde6g^CreERT2/+^* littermates at 7 and 12 dpi stained with phalloidin (red) and WGA (green). Phalloidin intensity along the white line is shown on the right side of the images. Arrows point to signal originating from an actin punctum at the base of an outer segment. Scale bars: 20 µm. Each image is a maximum intensity projection of five images taken at 0.7-µm intervals. (B) Enlargements of regions from A showing the actin puncta (arrows) at the junction between the connecting cilium and outer segment. Scale bars: 10 µm. Each image is a maximum intensity projection of five images taken at 0.7-µm intervals. (C) Confocal images of retinal sections of vehicle (control) and tamoxifen (experimental)-treated *Inpp5e^flox/flox^/Pde6g^CreERT2/+^* littermates 12 dpi stained with phalloidin (red) and for acetylated tubulin (Ac Tub, green). Each image is a maximum intensity projection of 20 images taken at 0.7-µm intervals. See Fig. S6 for red–green cone opsin staining of this image. (D) Fluorescence intensity of phalloidin puncta at the junction of the inner and outer segments in 12 dpi animals. *N* is 10–40 puncta measured from three animals per genotype. Each spot is one measurement. (E) Number of phalloidin puncta per linear µm at the inner-outer segment junction in 7 dpi and 12 dpi for vehicle- (control) and tamoxifen- (experimental) treated *Inpp5e^flox/flox^/Pde6g^CreERT2/+^* littermates. The number of phalloidin puncta were counted from images like in C and divided by the width of the image. *N*=3 animals per genotype. (F) Confocal images of retinal sections from vehicle (control) and tamoxifen (experimental)-treated *Inpp5e^flox/flox^/Pde6g^CreERT2/+^* littermates at 12 dpi stained with phalloidin (red) and for Wasf3 (green). Scale bars: 10 µm (A,C,F), 5 µm (B). OS, outer segment; IS, inner segment; ONL, outer nuclear layer; OPL, outer plexiform layer; INL, inner nuclear layer. Each image is a maximum intensity projection of two images taken at 0.7-µm intervals. (G) Number of Wasf3 and phalloidin puncta per linear micrometer at the inner–outer segment junction in 12 dpi animals. The number of Wasf3 and phalloidin puncta were counted from images as in A and divided by the width of the image. *N*=4 per genotype. (H) Fluorescence intensity of Wasf3 and phalloidin puncta at the junction of the inner and outer segments in 12 dpi animals. *N* is 40–50 puncta measured from three animals per genotype. Each spot is one measurement. In all panels, error bars represent s.d. *P<0.05; **P<0.01; ****P<0.0001; ns, not significant (unpaired two-tailed *t*-test).

The occasional membrane whorls emanating from the photoreceptor cilium in a subset of *Pde6g^CreERT2^/Inpp5e* mice (Fig. 1Cb,Db) resembles the overgrown discs observed in mice with disrupted actin network regulators (Spencer et al., 2019, 2023). To determine whether the actin cytoskeleton is disrupted by the loss of Inpp5e, we stained retinas with fluorescent phalloidin, which labels F-actin (Fig. 6; Fig. S5). In control retinas, phalloidin strongly stained the inner segment region with the most intense labeling just above the outer limiting membrane with staining extending into both the nuclear and inner segment layers. In addition, there was a small punctum at the outer segment base where new outer segment discs are formed. At 7 dpi, actin staining in *Pde6g^CreERT2^* experimental retinas looked similar to controls (Fig. 6A,B). However, at 12 dpi, the inner segment pool of F-actin was greatly depleted, except for a strong band lying just above the outer limiting membrane. This is the location of Muller glia, which have an extensive actin cytoskeleton (Tworig and Feller, 2021) and could be contributing to phalloidin signal in this region. Most actin puncta at the outer segment base were weaker in experimental animals at 12 dpi, but a few strong puncta remained (Fig. 6A,B). The latter puncta likely represent cones as they are located deeper in the inner segment layer and often mark the base of red–green opsin-positive outer segments (Fig. S6A). To quantify the intensity of the actin puncta at the base of the connecting cilia in 12 dpi animals, we co-stained with phalloidin and an acetylated tubulin antibody, which labels stable microtubules including axonemes (Fig. 6C). The number of axonemes in the mutants did not appear to be affected, but the intensity of the actin puncta associated with the axonemes in experimental animals was reduced to about a half of what was observed in controls (Fig. 6D), and the number of puncta were reduced at 12 dpi to about one fifth (Fig. 6E). Similar results were observed following *iCre75*-driven *Inpp5e* deletion, with inner segment labelling reduced at P15 and largely gone by P21, except for the band above the outer limiting membrane (Fig. S5).

The WASP-family verprolin-homologous protein (WAVE) complex interacts with Arp2/3 to drive branched actin formation (Takenawa and Suetsugu, 2007). In photoreceptors, the WAVE complex subunit Wasf3 serves as a crucial activator of Arp2/3 and is essential for nucleating actin polymerization that drives disc formation at the base of the outer segment (Corral-Serrano et al., 2020; Spencer et al., 2023). To determine whether the reduced phalloidin signal in the Inpp5e knockout was due to a failure of Wasf3 to localize properly, we co-stained retinal sections with phalloidin and anti-Wasf3 antibodies (Fig. 6F). Fewer phalloidin-positive and fewer Wasf3-positive puncta were observed in experimental animals lacking Inpp5e (Fig. 6G). However, the Wasf3-positive puncta were similar in intensity in both control and experimental animals, whereas phalloidin signal was reduced in the experimentals (Fig. 6H). This suggests that F-actin loss at the base of the outer segment is not a consequence of impairment of actin nucleation mediated by the WAVE complex.

## DISCUSSION

Human individuals with INPP5E pathogenic variants develop retinal degeneration (Bielas et al., 2009; Jacoby et al., 2009; Sangermano et al., 2021). Previous work using *Six3-Cre* to delete *Inpp5e* in the developing embryonic eye found that loss of Inpp5e largely blocked the formation of rod and cone outer segments and caused a rapid degeneration of the retina, such that almost no rod or cone cells remained at P21. Instead of forming outer segments, the axoneme failed to extend beyond the connecting cilium and the interior was filled with unorganized vesicles rather than membrane discs (Sharif et al., 2021). As this study removed Inpp5e prior to the photoreceptor cells becoming post-mitotic and initiating outer segment development, we sought to explore the role of Inpp5e in later stages of outer segment maturation and maintenance. In our study, we used *iCre75* to delete *Inpp5e* during the elongation phase of outer segment formation and the tamoxifen-inducible *Pde6g^CreERT2^* to delete *Inpp5e* from mature photoreceptors. Similar to what was observed previously with *Six3-Cre*, we found that the loss of *Inpp5e* from rod photoreceptor cells leads to the rapid degeneration of these cells. We found that loss of *Inpp5e* through *iCre75* or *Pde6g^CreERT2^* lead to shorter outer segments that lacked evaginating discs at the base. Our findings are consistent with the observation that no discs formed when *Inpp5e* was deleted through *Six3-Cre*. However, the plasma membrane of the outer segments in the *Six3-Cre* model appeared to have expanded without discs and this was not observed in our models.

The loss of Inpp5e was accompanied by accumulation of rhodopsin and, possibly, other proteins bound by WGA in the cell body, and the presence of extracellular vesicles containing rhodopsin. Although extracellular vesicles are occasionally seen in wild-type retinas (Lewis et al., 2024, 2022), their prevalence in our models resembled what is found in rhodopsin mutant models, as well as in other models characterized by rhodopsin mislocalization (Blanks et al., 1982; Blanks and Spee, 1992; Dilan et al., 2018; Gupta et al., 2018; Hagstrom et al., 1999; Heckenlively et al., 1995; Lewis et al., 2024, 2022; Marszalek et al., 2000; Pazour et al., 2002; Spencer, 2023). Recent work from Lewis and colleagues (Lewis et al., 2024) has found that extracellular vesicles released from photoreceptor cells degenerating due to *Ift20* deletion contain a high amount of rhodopsin. Interestingly, these extracellular vesicles have higher concentrations of rhodopsin than was found on the inner segment plasma membrane, suggesting that photoreceptors have a mechanism for concentrating mislocalized rhodopsin before releasing it from the cell. Whether the release of extracellular vesicles serves as a protective mechanism whereby cells discard excess rhodopsin to alleviate stress or the accumulation of vesicles in the extracellular environment is toxic to the retina remains to be identified in future studies (reviewed in Spencer, 2023).

Inpp5e was initially identified as a Golgi-associated enzyme (Kong et al., 2000). However, this localization has largely been overlooked in recent studies that focused on the ciliary role of the enzyme in regulating conversion of PI(4,5)P_2_ and PI(3,4,5)P_3_ into PI(4)P and PI(3,4)P_2_ (Dyson et al., 2017) to modulate Hedgehog signaling (Chávez et al., 2015; Constable et al., 2020; Garcia-Gonzalo et al., 2015) and ciliary disassembly (Phua et al., 2017). In the retina, we found that the loss of the loss of Inpp5e leads to vacuolation and morphological changes in the Golgi apparatus suggesting that the Golgi pool of Inpp5e is likely important to the pathology that results from its loss. In addition, we observed elevated rhodopsin and WGA staining in the inner segment, supporting a role for Inpp5e in the Golgi complex or in trafficking within the inner segment.

Phalloidin staining of the actin cytoskeleton was reduced with *Inpp5e* deletion. Phosphoinositides are well known regulators of actin dynamics. Of relevance to our work, the Inpp5e substrates PI(4,5)P_2_ and PI(3,4,5)P_3_ are thought to promote actin filament assembly (Hilpela et al., 2004; Janmey et al., 2018). A simple model would suggest that the loss of Inpp5e would elevate these phosphoinositides promoting actin polymerization. However, this was not observed, suggesting a more complicated role for Inpp5e. Although the mechanism is unknown, it is possible that indirect effects due to the Golgi dysfunction or trafficking abnormalities drive the observed cytoskeletal defects.

## MATERIALS AND METHODS

### Mice

The handling of mice in this study was approved by the institutional animal care and use committees of the UMass Chan School of Medicine. The mice were housed under a 12-h light and 12-h dark cycle and had unrestricted access to food and water. Wild-type C57BL6/J mice were obtained from Jackson Labs (000664, Bar Harbor ME, USA). *Inpp5e^flox/flox^* mice (Dyson et al., 2017) were obtained from Christina Mitchell at Monash University, Australia. *Pde6g^CreERT2^* mice (Koch et al., 2015) were obtained from Stephen Tsang of Columbia University, USA. The *iCre75* mice (Li et al., 2005) were obtained from Jackson Labs (015850). Genotyping was performed by PCR using the primers in Table S1. None of the mice carried the *Pde6b^rd1^* or *Crb1rd^8^* retinal degeneration alleles.

For inducible deletion of *Inpp5e* in rod photoreceptor cells through *Pde6g^CreERT2^*, 100 µg of tamoxifen (T5648-1G, Sigma-Aldrich, St Louis MO, USA) per gram of body weight was injected intraperitoneally for four consecutive days. Littermates were used as controls.

### Electroretinography

Electroretinogram (ERG) recordings were conducted using a Celeris system (Diagnosys, Lowell MA, USA) to evaluate retinal responses under combined dark- and light-adapted conditions, including a flicker response. After a 12-h dark adaptation period, mice were anesthetized via an intraperitoneal injection of a ketamine-xylazine mixture (100 mg/kg of body weight and 10 mg/kg of body weight, respectively). Pupil dilation was achieved by applying one drop each of phenylephrine (2.5%) and tropicamide (1%) at ∼10 min before recording. Throughout the ERG procedure, animals were maintained on a warming plate to sustain a body temperature of 37°C. ERG electrodes were directly positioned on the eyes and a flicker ERG frequency series was performed with intensities of 0.01 cd·s/m^2^, 0.1 cd·s/m^2^, and 1 cd·s/m^2^, to capture the rod response. Subsequently, a 10-min light adaptation phase was initiated, followed by recording cone impulse responses at intensities of 3 cd·s/m^2^ and 10 cd·s/m^2^, as well as a 10 Hz flicker response. The ERG waveform data were assessed for amplitude and latency of response components, including flicker responses.

### Immunofluorescence microscopy

Mice were anesthetized by CO_2_ asphyxiation, their eyes removed and placed in 4% paraformaldehyde in Sorensen buffer (100 mM phosphate, pH 7.2) overnight at 4°C. After fixation, the eyecups were dissected and embedded in 2.5% low-melt agarose (A3038, Sigma-Aldrich). The agarose-embedded eyes were sliced into 100-µm thick sections using a vibratome (VT1200S, Leica, Deer Park IL, USA) (Lobanova et al., 2010; Spencer et al., 2019).

Sections for immunostaining were blocked in Sorensen buffer containing 7% goat serum (ab7481, Abcam, Waltham, MA, USA) and 0.5% Triton X-100 (18607596, Thermo Fisher Scientific, Pittsburgh, PA, USA) for 1 h at room temperature followed by 30 min incubation with antibodies, lectins and toxins (Table S2). Sections were washed three times and incubated with secondary antibodies for 2 h at room temperature (Table S2). Following staining, sections were washed three times with PBS and mounted on slides (P36931, Invitrogen, Waltham MA, USA).

To prepare cryosections, mouse eyes were fixed in 4% paraformaldehyde in Sorensen buffer for 10 min and then transferred to phosphate buffer without fixative. For cryoprotection, the eyes were sequentially immersed in phosphate buffer supplemented with 10%, 20%, and 30% sucrose for 1 h, 4 h, and overnight, respectively. Subsequently, the eyes were embedded in an optical cutting temperature compound (4583 Tissue-Tek OCT, Sakura, Torrance CA, USA) and frozen on dry ice. Using a cryostat, 10-μm sections were cut from the frozen embedded eyes. Sections were blocked in 5% normal goat serum and 0.1% Triton X-100 in Sorensen buffer for 1 h. Primary and secondary antibodies (Table S2) diluted in blocking buffer were incubated overnight at 4°C and 1 h at room temperature respectively. After washing nuclei were stained with 10 µg/ml DAPI for 15 min at room temperature and the tissue mounted with ProLong Gold Antifade Mountant with DAPI (P36931, Vectorlabs, Newark CA, USA).

To quantify Golgi morphology, images were analyzed using Fiji/ImageJ software. Ellipticity of Golgi structures was determined by measuring the length of the major and minor axes using the line tool. The major axis was defined as the longest continuous dimension of the Golgi structure. The minor axis was measured as the longest dimension perpendicular to the major axis. Ellipticity was calculated as the ratio of major to minor axis lengths. A minimum of three individual Golgi structures were measured from three different fields per sample, with three biological replicates per condition.

### Transmission electron microscopy

Mice were deeply anesthetized and transcardially perfused with 2% paraformaldehyde, 2% glutaraldehyde and 0.05% calcium chloride in 50 mM MOPS buffer (pH 7.4) (Ding et al., 2015). Subsequently, the eyes were removed and fixed for an additional 2 h in the same buffer at 22°C. After washing in PBS, the eyecups were dissected and embedded in 5% agar (A3038, Sigma-Aldrich) in PBS. 200-µm-thick sections were obtained with a vibratome (VT1200S, Leica, Deer Park IL, USA). The vibratome sections were stained with 1% tannic acid and 1% uranyl acetate (21700 and 22400, Electron Microscopy Sciences, Hatfield PA, USA), dehydrated with ethanol, and then infiltrated and embedded in Spurr resin (02680-AB, SPI Supplies, West Chester, PA, USA). 70-nm-thick sections were cut and placed on copper grids. The sections were counterstained with 2% uranyl acetate and 3.5% lead citrate (19481 and 19314, Ted Pella, Redding CA, USA) to enhance contrast. Sections were imaged using either a Philips CM10 electron microscope at 100 kV accelerating voltage with a Gatan TEM CCD camera or a JEOL JEM-1400 electron microscope at 60 kV with an AMT BioSprint camera.

### Statistics

Data groups were analyzed as described in the figure legends using Prism (GraphPad, San Diego CA, USA) using unpaired two-tailed *t*-tests. Differences between groups were considered statistically significant if *P*<0.05. Statistical significance is indicated with asterisks (*P=0.01–0.05, **P=0.01-0.001, ***P=0.001-0.0001, ****P<0.0001). Error bars are standard deviation (s.d.).

## Supplementary Material



10.1242/joces.263814_sup1 Supplementary information
