# Supplementary material for: Inpp5e is crucial for photoreceptor outer segment maintenance
Source: J Cell Sci. 2025 Feb 21;138(4):JCS263814. doi: 10.1242/jcs.263814 (PMC11883294; doi:10.1242/jcs.263814)
Supplement: Supplementary information [file joces-138-263814-s1.pdf]

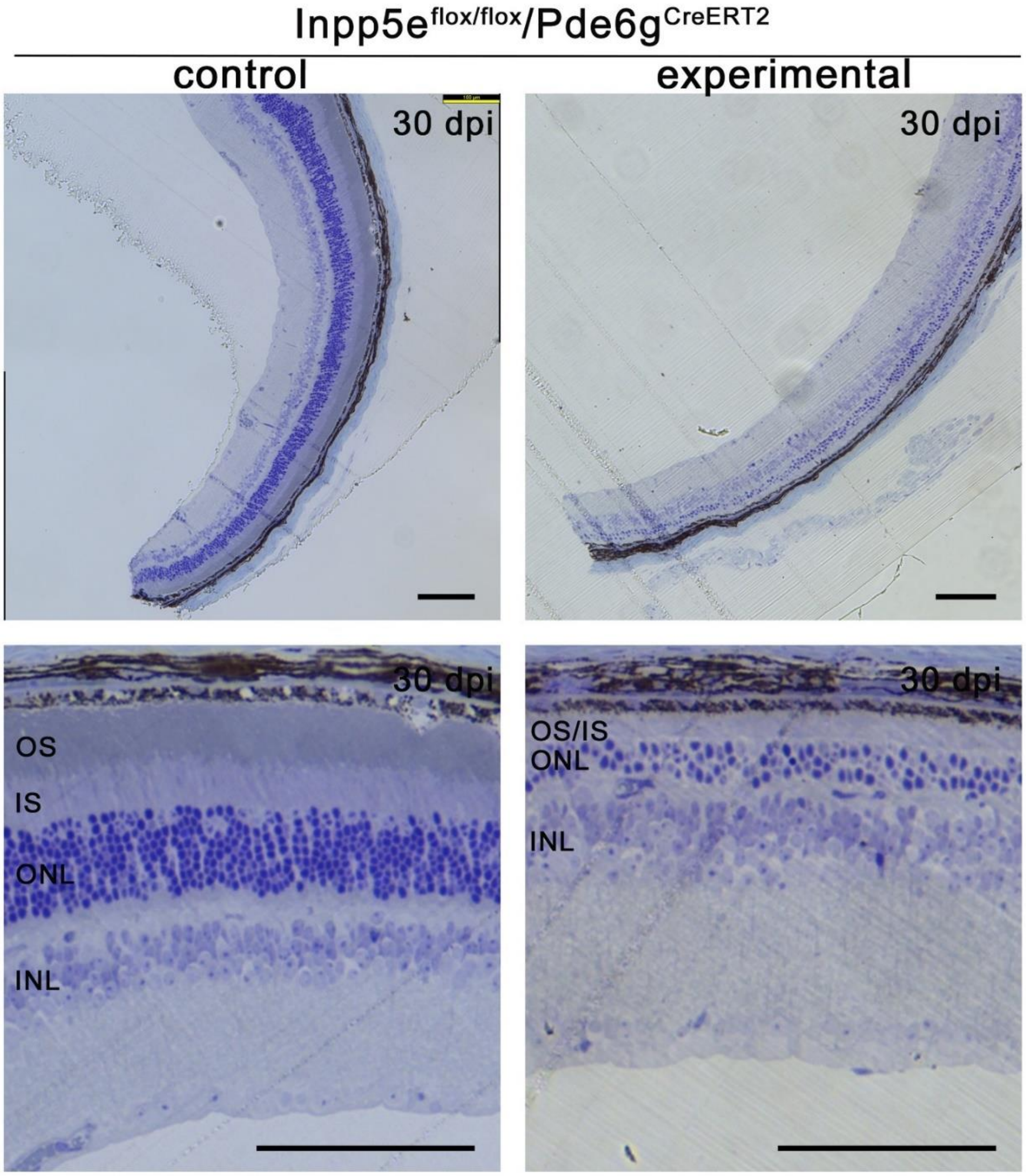

Fig. S1. Loss of Inpp5e leads to severe photoreceptor degeneration.

Light microscopy of toluidine blue-stained retinal sections from *Inpp5e*$^{flox/flox}$/*Pde6g*^{CreERT2} littermates treated with vehicle (control) or tamoxifen (experimental) and examined at 30 days post last injection. Experimental retinas show severe thinning of the outer nuclear layer (ONL) and near-complete loss of outer segments. OS, outer segment; IS, inner segment; ONL, outer nuclear layer; INL, inner nuclear layer. Scale bar 100 µm.

Journal of Cell Science • Supplementary information

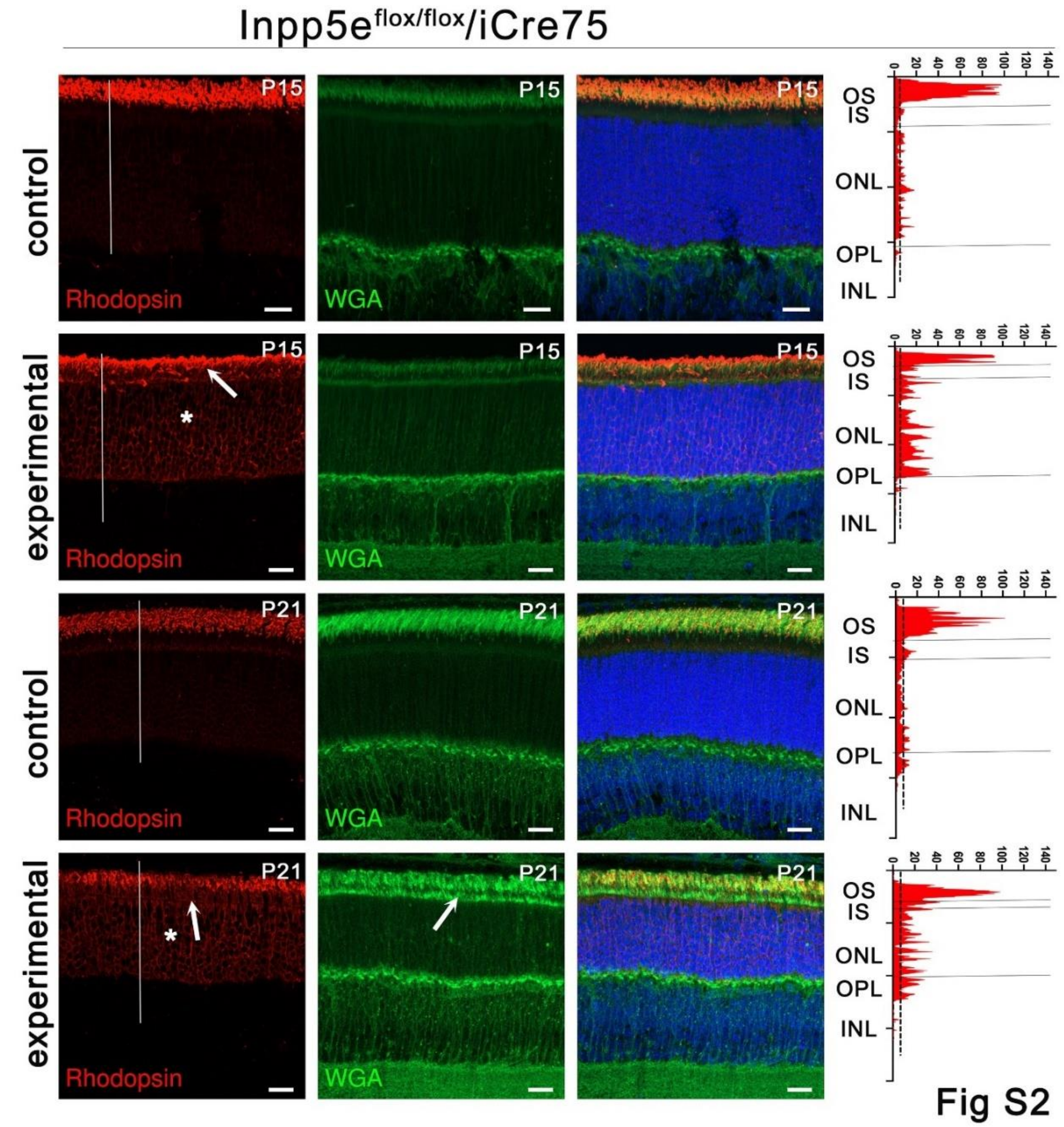

Fig. S2. ***iCre75*-driven loss of Inpp5e causes rhodopsin mislocalization.**

(A) Confocal images of retinal sections (agarose embedded) of *Inpp5e$^{flox/flox}$* (control) and *Inpp5e$^{flox/flox}$/iCre75* (experimental) littermates at P15 and P21 immunostained with anti-rhodopsin clone 4D2 (red) and wheat germ agglutinin (WGA, green). Scale bar: 20 µm. Each image is a maximum intensity projection of 20 images taken at 0.7-µm intervals. Rhodopsin intensity along the white line is shown on the right side of the images. Arrows point to mislocalized rhodopsin or WGA in the inner segment. * points to rhodopsin in the nuclear layer. OS, outer segment; IS, inner segment; ONL, outer nuclear layer; OPL, outer plexiform layer; INL, inner nuclear layer.

Journal of Cell Science • Supplementary information

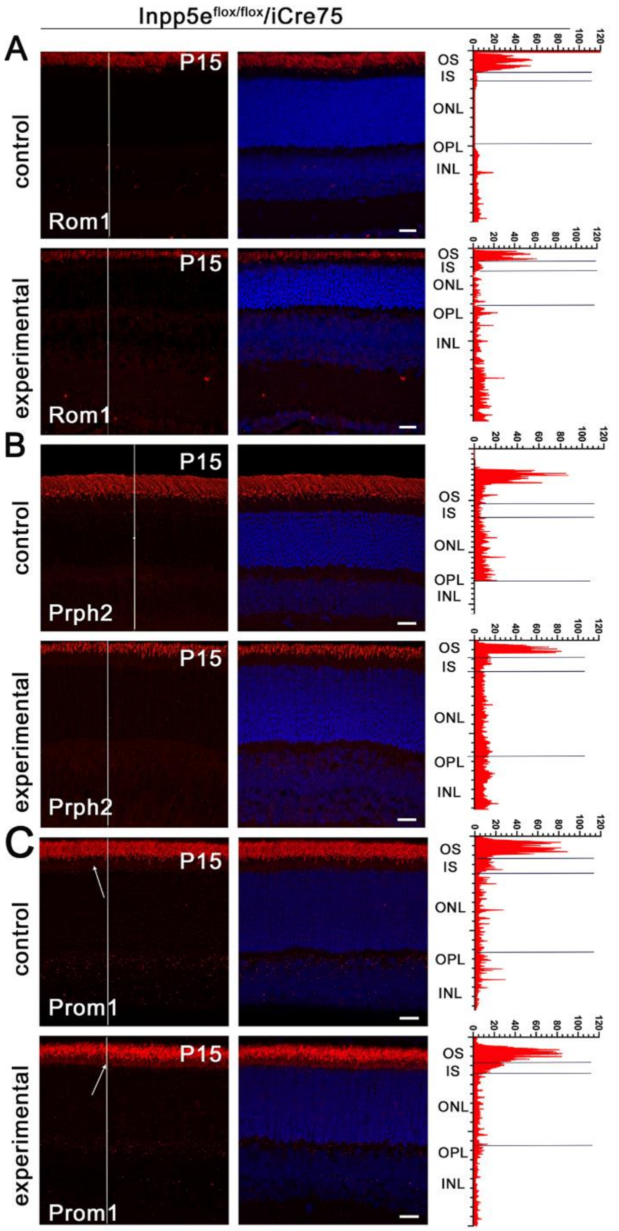

Fig S3

Fig. S3. Loss of Inpp5e alters distribution of Prom1 in the *iCre75* cohort.

(A-C) Confocal images of retinal sections of *Inpp5e*$^{flox/flox}$ (control) and *Inpp5e*$^{flox/flox}$*/iCre75* (experimental) littermates examined at P15. Sections were stained with DAPI (blue) and for Rom1 (A), Prph2 (B), and Prom1 (C) in red. Note increased Prom1 in the inner segments (arrow) of the experimental animals compared to controls. Scale bars are 20 µm. Each image is a maximum intensity projection of 20 images taken at 0.7-µm intervals. The intensity of the red channel along the white line is shown on the right side of the images. OS, outer segment; IS, inner segment; ONL, outer nuclear layer; OPL, outer plexiform layer; INL, inner nuclear layer.

Journal of Cell Science • Supplementary information

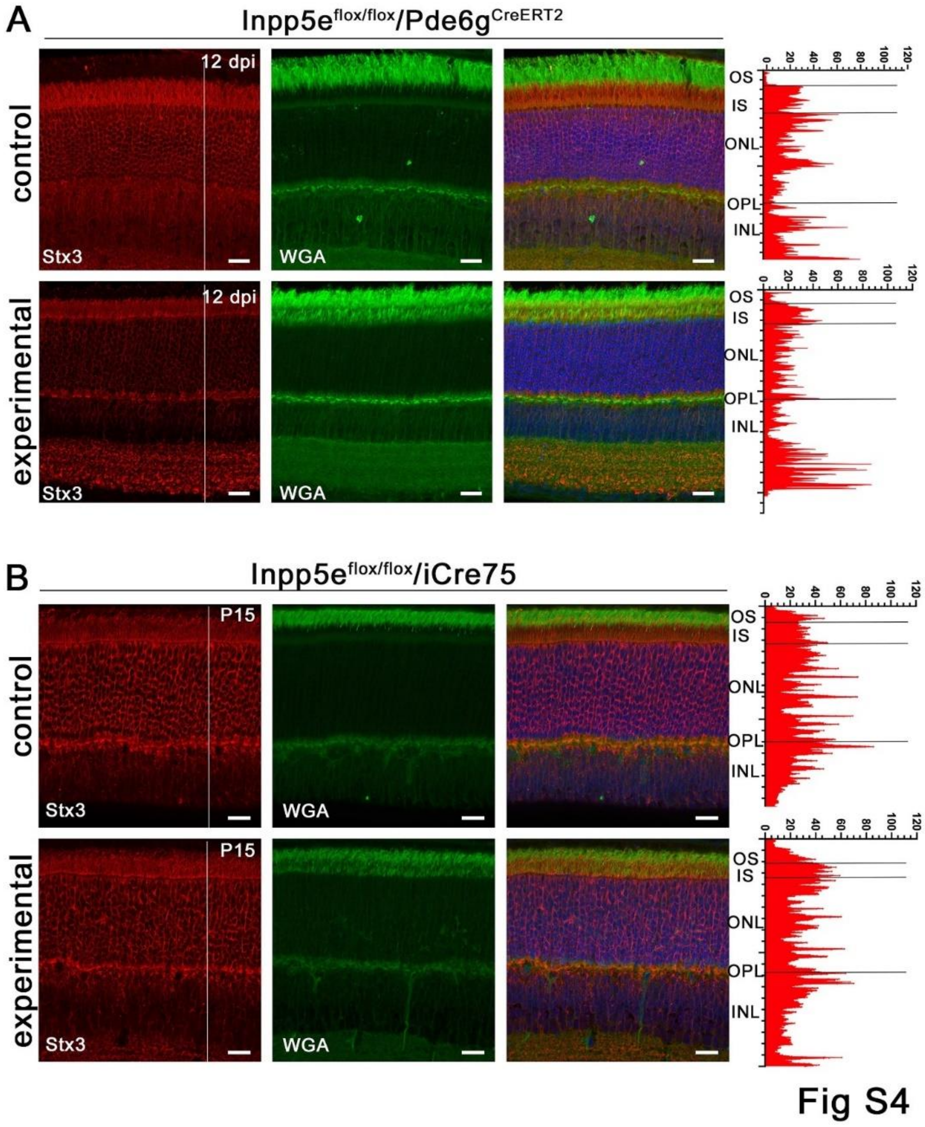

Fig. S4. Stx3 is not mislocalized by Inpp5e loss.

(A-B) Confocal images of retinal sections of vehicle- (control) and tamoxifen- (experimental) treated *Inpp5e*$^{flox/flox}$/*Pde6g*^{CreERT2} littermates at 12 days post last injection (dpi) (A) or *Inpp5e*$^{flox/flox}$ (control) and *Inpp5e*$^{flox/flox}$/*iCre75* (experimental) littermates examined at P15 (B). Sections were stained with DAPI (blue), wheat germ agglutinin (WGA, green) and syntaxin-3 (Stx3, red). Scale bars are 20 µm. Each image is a maximum intensity projection of 20 images taken at 0.7-µm intervals. The intensity of the red channel along the white line is shown on the right side of the images. OS, outer segment; IS, inner segment; ONL, outer nuclear layer; OPL, outer plexiform layer; INL, inner nuclear layer.

Journal of Cell Science • Supplementary information

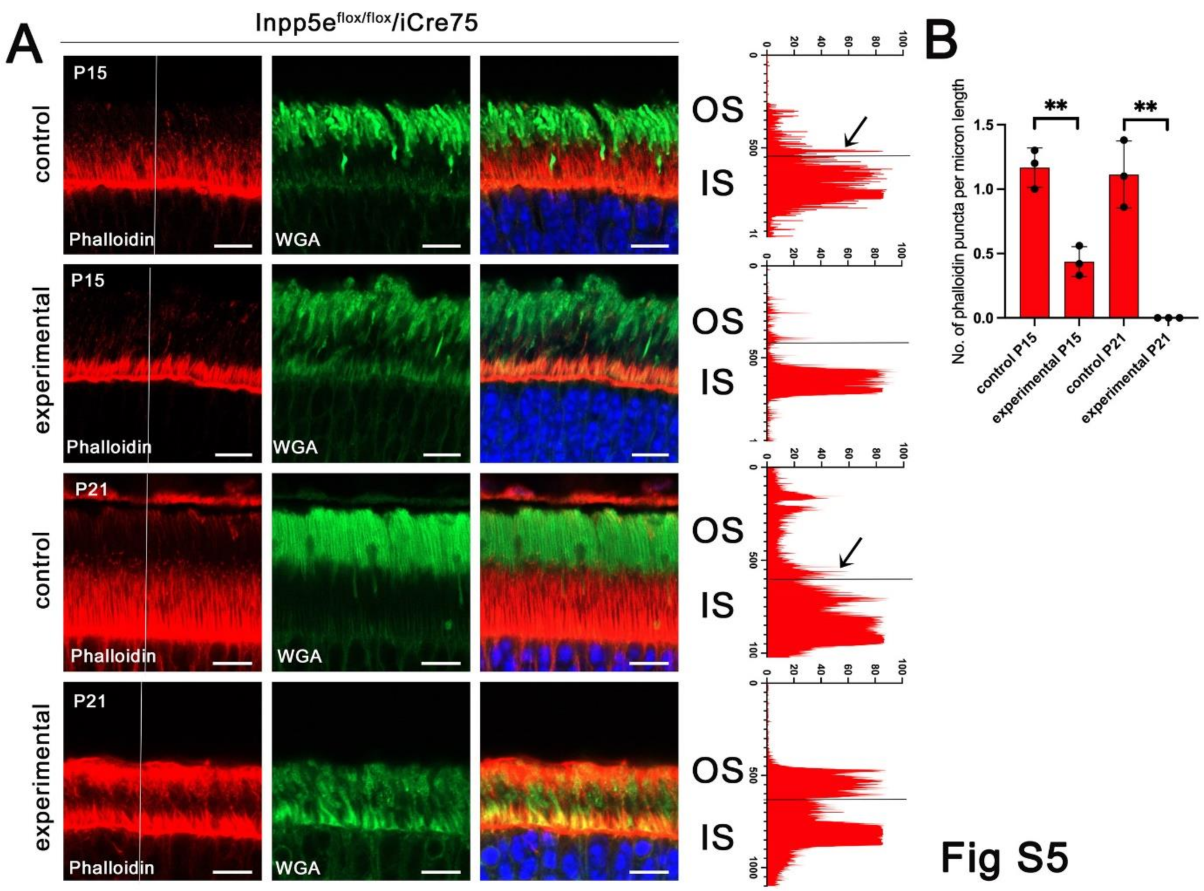

Fig. S5. *iCre75*-driven loss of Inpp5e disrupts the actin cytoskeleton.

(A) Confocal images of retinal sections of *Inpp5e*$^{flox/flox}$ (control) and *Inpp5e*$^{flox/flox}$*/iCre75* (experimental) littermates at P15 and P21 stained with phalloidin (red), and wheat germ agglutinin (WGA, green). Phalloidin intensity along the white line is shown on the right side of the images. Arrows point to signal originating from an actin punctum at the base of an outer segment. Scale bar: 20 µm. Each image is a maximum intensity projection of 2 images taken at 0.7-µm intervals.

(B) Number of phalloidin puncta per linear µm at the inner-outer segment junction in P15 and P21 animals. The number of phalloidin puncta were counted from images like in A and divided by the width of the image. N = 3 animals per genotype. **$p<0.01$, by unpaired t-test.

Journal of Cell Science • Supplementary information

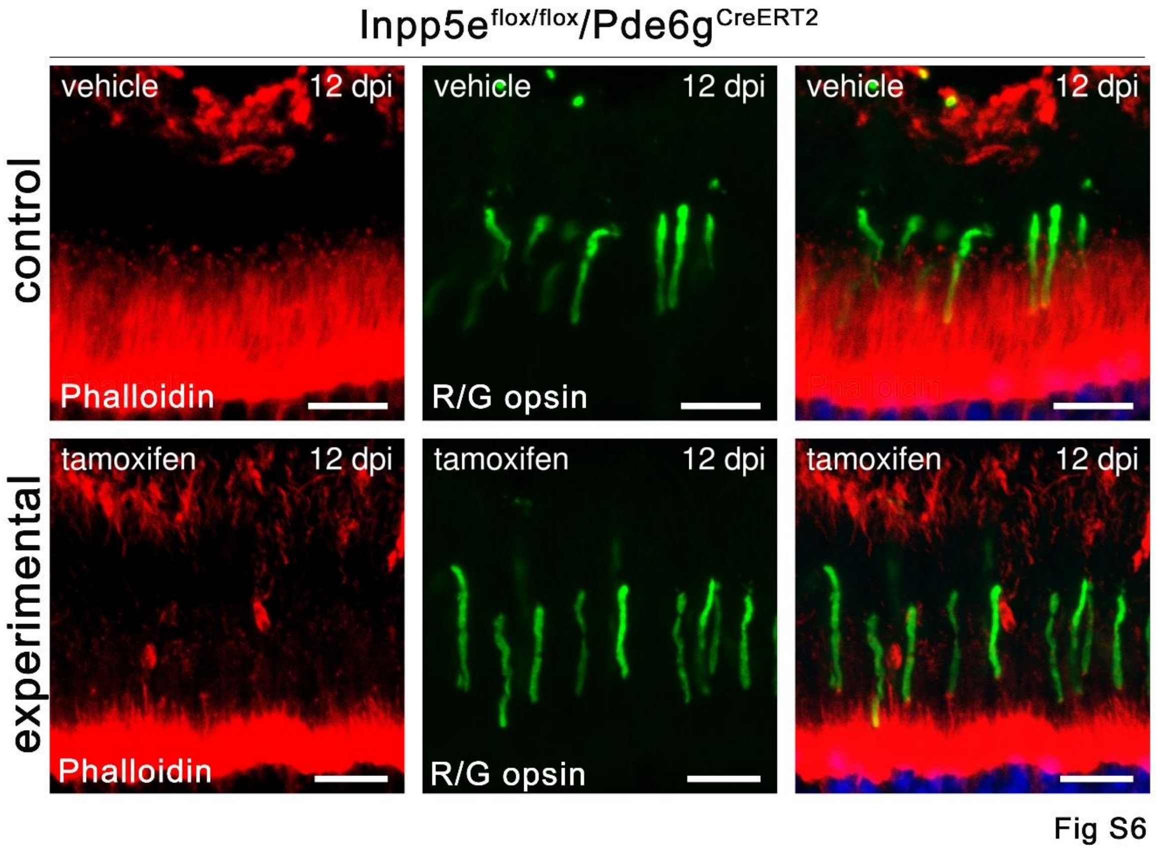

Fig. S6. Actin puncta remain at the base of cones.

Confocal images of retinal sections of vehicle- (control) and tamoxifen- (experimental) treated *Inpp5e$^{flox/flox}$/Pde6g$^{CreERT2/+}$* littermates at 12 days post last injection (dpi) stained with phalloidin (red), and red/green opsin (R/G opsin, stained with Alexa647 and pseudocolored green). Scale bars:10 µm. Each image is a maximum intensity projection of 8 images taken at 0.7-µm intervals. See Figure 6 for a phalloidin and acetylated tubulin labeled version of this image.

Journal of Cell Science • Supplementary information

Table S1. Genotyping primers

Primer	Sequence	Product
Inpp5e-floxF<br>Inpp5e-floxR	GAGAAGCTGATAGATGGCTAGG<br>AACCAGAAGACCTCATCAAACC	flox: 418bp<br>WT: 300 bp
Pde6g-wtF<br>Pde6g-creR	GGTCAGATTCCAGTGTGTGGG<br>GTTTAGCTGGCCCAAATGTTG	$Pde6g^{CreERT2}$: 715bp<br>WT: none
Pde6g-wtF<br>Pde6g-wtR	GGTCAGATTCCAGTGTGTGGG<br>CTTAGGTGGTCCTTTCCTGGG	$Pde6g^{CreERT2}$: none<br>WT: 514 bp
iCre75-F-MG<br>iCre75-R-MG	GCATTGCCGAAATTGCCAGA<br>GGCAGCCACACCATTCTTTC	iCre75: 234 bp<br>WT: none
Rd1-wt<br>Rd1-common	ACCTGCATGTGAACCCAGTATTCTATC<br>CTACAGCCCCTCTCCAAGGTTTATAG	*Pde6b*^{rd1}: none<br>WT: 240 bp
Rd1-mt<br>Rd1-common	AAGCTAGCTGCAGTAACGCCATTT<br>CTACAGCCCCTCTCCAAGGTTTATAG	*Pde6b*^{rd1}: 560 bp<br>WT: none
Rd8-wtF<br>Rd8-wtR	GTGAAGACAGCTACAGTTCTGATC<br>GCCCCATTTGCACACTGATGAC	*Crb1*^{rd8}: none<br>WT: 220 bp
Rd8-mtF<br>Rd8-wtR	GCCCCTGTTTGCATGGAGGAAACTTGGA AGACAGCTACAGTTCTTCTG<br>GCCCCATTTGCACACTGATGAC	*Crb1*^{rd8}: 244 bp<br>WT: none

Journal of Cell Science • Supplementary information

Table S2. Antibodies, Lectins, and Toxins

	Identifier	Supplier	Concentration
**Antibody**			
Inpp5e	17797-AP	Proteintech, Rosemont IL USA	1:200
Rhodopsin	Ab98887 (4D2)	Abcam, Cambridge UK	1:2000
Red/Green Opsin	AB5405	MilliporeSigma, Burlington, MA USA	1:2000
Ift27	719	(Keady et al., 2012)	1:1000
Ift88	448	(Pazour et al., 2002)	1:1000
Ift140	964	(Jonassen et al., 2012)	1:1000
Cep164	22227-1AP	Santa Cruz Biotech, Santa Cruz CA USA	1:1000
Prph2	RDS-CT	(Stuck et al., 2014)	1:2000
Rom1		(Spencer et al., 2023)	1:5000
Prom1	MAB4310	MilliporeSigma, Burlington, MA USA	1:100
Stx3	15556-1-AP	Proteintech, Rosemont IL USA	1:2000
Wasf3	2806S	Cell Signaling Technology	1:500
Giantin	3991	(Nozawa et al., 2002)	1:200
Acetylated tubulin	611-B1 (MABT888)	Sigma, St Louis MO USA	1:2000
Anti-mouse-Alexa488<br>-Alexa594<br>-Alexa647	A-11017<br>A-11032<br>A-21235	Invitrogen, Waltham, MA USA	1:1000
Anti-rabbit-Alexa488<br>-Alexa594<br>-Alexa647	A-11034<br>A-21207<br>A-21245	Invitrogen, Waltham, MA USA	1:1000
**Lectins, toxins, etc.**			
Wheat germ agglutinin (WGA)-Alexa488	W11261	Invitrogen, Waltham, MA USA	1 µg/mL
Phalloidin-Alexa594	A12381	Invitrogen, Waltham, MA USA	1:500
4',6-diamidino-2-phenylindole (DAPI)	D1306	Invitrogen, Waltham, MA USA	10 µg/ml

Keady, B. T., Samtani, R., Tobita, K., Tsuchya, M., San Agustin, J. T., Follit, J. A., Jonassen, J. A., Subramanian, R., Lo, C. W. and Pazour, G. J. (2012). IFT25 links the signal-dependent movement of Hedgehog components to intraflagellar transport. *Dev Cell* **22**, 940-51.

Journal of Cell Science • Supplementary information

Nozawa, K., Casiano, C. A., Hamel, J. C., Molinaro, C., Fritzler, M. J. and Chan, E. K. (2002). Fragmentation of Golgi complex and Golgi autoantigens during apoptosis and necrosis. *Arthritis Res* **4**, R3.

Pazour, G. J., Baker, S. A., Deane, J. A., Cole, D. G., Dickert, B. L., Rosenbaum, J. L., Witman, G. B. and Besharse, J. C. (2002). The intraflagellar transport protein, IFT88, is essential for vertebrate photoreceptor assembly and maintenance. *J Cell Biol* **157**, 103-13.

Spencer, W. J., Schneider, N. F., Lewis, T. R., Castillo, C. M., Skiba, N. P. and Arshavsky, V. Y. (2023). The WAVE complex drives the morphogenesis of the photoreceptor outer segment cilium. *Proc Natl Acad Sci U S A* **120**, e2215011120.

Stuck, M. W., Conley, S. M. and Naash, M. I. (2014). The Y141C knockin mutation in RDS leads to complex phenotypes in the mouse. *Hum Mol Genet* **23**, 6260-74.

Journal of Cell Science • Supplementary information
