# Supplementary material for: Inpp5e is crucial for photoreceptor outer segment maintenance
Source: J Cell Sci. 2025 Feb 21;138(4):JCS263814. doi: 10.1242/jcs.263814 (PMC11883294; doi:10.1242/jcs.263814)
Supplement: Supplementary file 1 [file joces-138-263814_review_history.pdf]

Inpp5e is crucial for photoreceptor outer segment maintenance

Mohona Gupta, Taylor R. Lewis, Michael W. Stuck, William J. Spencer, Natalia V. Klementieva, Vadim Y. Arshavsky and Gregory J. Pazour

DOI: 10.1242/jcs.263814

Editor: Michael Way

Review timeline

Submission to Review Commons:	28 August 2024
Submission to Journal of Cell Science:	24 December 2024
Accepted:	7 January 2025

Reviewer 1

Evidence, reproducibility and clarity

This manuscript provides a partial phenotypic description of knockouts of the Inpp5e gene in the mouse retina at different developmental timepoints: one during the initial expression of rhodopsin in rods and emergence of outer segments (ICre75), and the other after full development of many rod outer segments and 12 days after administration of a knockout-inducing drug (tamoxifen). As far as it goes, the phenotypic characterization seems to be technically sound, although it is carried out at a limited number of time points. The question arises, since this gene has been inactivated previously in mouse retina, "what valuable new information is provided by the present work?" One item is the conclusion that this protein is not only essential for early development of outer segments (already known), but also for renewal of outer segments after rod maturation (new).
It is somewhat surprising that there is a detectable b-wave as late as P30 in the iCre75 retinas, suggesting that at least some semblance of outer segments may have developed and survived up to this time point without INPP5E protein. However, that conclusion is rendered somewhat ambiguous by the uncertainty of the lifetime of whatever pool of INPP5E was already present prior to gene inactivation in the later stages of rod development. This caveat is underscored by the observation of a few OS with newly formed discs in the CreERT2 mice 12 days after tamoxifen administration, possibly due to a small remaining pool of functional INPP5E at this time point. This is a point the authors may want to discuss.

Perhaps the most interesting new observation is the large accumulation of extracellular vesicles in the CreERT2 model, a phenomenon seen previously in retinas of mice with defects in ciliary function or structure. Because these were not observed in the previously published model (although intracellular vesicles were observed) it would be especially interesting to know if these are also formed in the ICre75 mice, but no data are provided to answer that question.

It would not be surprising if these vesicles contain rhodopsin, given its high abundance and the fact that trafficking to OS is largely blocked. However, the immuno-EM data presented are not particularly convincing. There are some black dots on the vesicles, but there are lots of black dots, some colored and some not. At the least, the Methods should describe what criteria were used to distinguish pixels corresponding to antibody staining from others of similar appearance.

The immunofluorescence evidence for disruption of Golgi is not particularly strong. A single image each is shown for control and knockout cells, and a single antigen was imaged and the images have low signal-to-noise. Despite the assignment of 4 asterisks to the difference between control and experimental measurements in Fig. 4C, the difference in means is small and the noise is high, without, apparently even taking into account the intrinsic noise in the signal. If such data are to

© 2025. Published by The Company of Biologists under the terms of the Creative Commons Attribution License (https://creativecommons.org/licenses/by/4.0/).

be taken seriously and conclusions drawn from them, details of the image analysis and statistical treatment, which are missing, should be provided in the Methods.

Finally, the death of cells is not well documented. In the earlier paper such death was obvious, but here there is only documentation of the loss of proper OS structure and some distortion of internal membranes in the IS. It seems likely that loss of OS and rhodopsin mislocalization ultimately would lead to extensive, if not complete, loss of rod nuclei but no evidence is provided that bears on that hypothesis. This weakness is somewhat disappointing, considering the technical feasibility of such experiments, once the animals are in hand. Although the manuscript refers to "rapid degeneration" it is not obvious that much cell death has occurred at the time points sampled.

Referees cross-commenting

I think it should not take too long for the authors to address the concerns raised by all three reviewers, who seem to agree that the manuscript is of value but has some weaknesses.

Significance

This manuscript provides a partial phenotypic description of knockouts of the Inpp5e gene in the mouse retina at different developmental timepoints: one during the initial expression of rhodopsin in rods and emergence of outer segments (ICre75), and the other after full development of many rod outer segments and 12 days after administration of a knockout- inducing drug (tamoxifen). As far as it goes, the phenotypic characterization seems to be technically sound, although it is carried out at a limited number of time points. The question arises, since this gene has been inactivated previously in mouse retina, "what valuable new information is provided by the present work?" One item is the conclusion that this protein is not only essential for early development of outer segments (already known), but also for renewal of outer segments after rod maturation (new).
It is somewhat surprising that there is a detectable b-wave as late as P30 in the iCre75 retinas, suggesting that at least some semblance of outer segments may have developed and survived up to this time point without INPP5E protein. However, that conclusion is rendered somewhat ambiguous by the uncertainty of the lifetime of whatever pool of INPP5E was already present prior to gene inactivation in the later stages of rod development. This caveat is underscored by the observation of a few OS with newly formed discs in the CreERT2 mice 12 days after tamoxifen administration, possibly due to a small remaining pool of functional INPP5E at this time point. This is a point the authors may want to discuss.

Perhaps the most interesting new observation is the large accumulation of extracellular vesicles in the CreERT2 model, a phenomenon seen previously in retinas of mice with defects in ciliary function or structure. Because these were not observed in the previously published model (although intracellular vesicles were observed) it would be especially interesting to know if these are also formed in the ICre75 mice, but no data are provided to answer that question.

It would not be surprising if these vesicles contain rhodopsin, given its high abundance and the fact that trafficking to OS is largely blocked. However, the immuno-EM data presented are not particularly convincing. There are some black dots on the vesicles, but there are lots of black dots, some colored and some not. At the least, the Methods should describe what criteria were used to distinguish pixels corresponding to antibody staining from others of similar appearance.

The immunofluorescence evidence for disruption of Golgi is not particularly strong. A single image each is shown for control and knockout cells, and a single antigen was imaged and the images have low signal-to-noise. Despite the assignment of 4 asterisks to the difference between control and experimental measurements in Fig. 4C, the difference in means is small and the noise is high, without, apparently even taking into account the intrinsic noise in the signal. If such data are to be taken seriously and conclusions drawn from them, details of the image analysis and statistical treatment, which are missing, should be provided in the Methods.
Finally, the death of cells is not well documented. In the earlier paper such death was obvious, but here there is only documentation of the loss of proper OS structure and some distortion of

© 2025. Published by The Company of Biologists under the terms of the Creative Commons Attribution License (https://creativecommons.org/licenses/by/4.0/).

internal membranes in the IS. It seems likely that loss of OS and rhodopsin mislocalization ultimately would lead to extensive, if not complete, loss of rod nuclei but no evidence is provided that bears on that hypothesis. This weakness is somewhat disappointing, considering the technical feasibility of such experiments, once the animals are in hand. Although the manuscript refers to "rapid degeneration" it is not obvious that much cell death has occurred at the time points sampled.

Reviewer 2

Evidence, reproducibility and clarity

This study examined the role of INPP5E in transciliary trafficking in retinal photoreceptors. Human patients with INPP5E pathogenic variants develop retinal degeneration. While the role of INPP5E in ciliary trafficking have been reported before, the current study utilized tissue specific and inducible promoter constructs to inactivate INPP5E at both the onset of photoreceptor outer segment morphogenesis and in mature photoreceptors, thus providing a fuller view of the role of INPP5E in retinal photoreceptors. The study finds a slew of cell biological defects including rhodopsin mis-localization to the inner segments, malformation of disc membranes and disruption of actin filament networks that are thought to help extend nascent discs at the base of outer segments.

Major comment: The study is well designed and carried out. The data are of high quality and support their conclusions. It provides new insights to cilia biology and function of INPP5E.

Minor comment: Page 3, 5th line from bottom: PI(4)P2 should be PI(4)P.

Significance

The study contributes new knowledge to the pathophysiological process related to INPP5E mutations, although it does not examine these issues at the molecular detail.

The manuscript is of interest to researchers who are generally interested in retinal degeneration, cilia biology and ciliopathy.

Reviewer 3

Evidence, reproducibility and clarity

Summary

This manuscript investigates the role of inositol polyphosphate-5-phosphatase E (INPP5E) in the development and maintenance of photoreceptors. Utilizing two distinct animal models, the authors examine the effects of INPP5E knockout in rod photoreceptors during two critical developmental stages: early retinal development, when photoreceptors are post-mitotic, and post-developmental stages. The findings reveal that the absence of INPP5E results in: 1) diminished rod photoresponse, 2) failure of nascent photoreceptor disc formation, 3) mislocalization of rhodopsin with subsequent accumulation of rhodopsin-containing vesicles near the inner segments, 4) structural disruption of the Golgi apparatus, and 5) alterations to the actin cytoskeleton, as evidenced by F-actin staining. The authors conclude that the observed reduction in photoresponse is likely attributed to the failure of photoreceptors to develop new outer segments and that INPP5E plays a critical role in protein trafficking.

Major comments on the article

I recommend that the authors include Western blot analysis and/or immunostaining for INPP5E in the knockout models to validate the effectiveness of the genetic manipulations. Additionally, the immunohistochemical (IHC) images demonstrating Prom1 mislocalization do not convincingly

© 2025. Published by The Company of Biologists under the terms of the Creative Commons Attribution License (https://creativecommons.org/licenses/by/4.0/).

support the claim. To strengthen this aspect, it would be beneficial to provide multiple images from various retinal regions. additionally, the study by Sharif et al. (2021) indicates that Prom1 does not mislocalize in the INPP5E knockout using the Six3Cre line; while the authors acknowledge this discrepancy, they do not offer a potential explanation.
Another point to consider is that there could be additional markers used to confirm Golgi dysfunction as well as disruption of the F-actin network. Particularly, additional evidence for F-actin network disruption should be considered given that the Wasf3 staining looked similar between the experimental and control mice.

Are the claims and the conclusions supported by the data, or do they require additional experiments or analyses to support them?

The claims presented are largely supported by the data; however, the assertion that INPP5E is necessary for protein trafficking could benefit from further clarification. It remains unclear whether this necessity stems from a direct interaction with specific proteins or is a consequence of previously established impairments in axoneme extension and abnormal localization of intraflagellar transport (IFT) particles observed in INPP5E knockout models.

Do you have constructive further-reaching suggestions that could significantly improve the study but would open new lines of investigation? Please label them as "OPTIONAL".

Are the suggested experiments realistic in terms of time and resources? It would help if you could add an estimated time investment for substantial experiments.

Yes, the proposed experiments appear realistic regarding time and resource requirements. Are the data and the methods presented in such a way that they can be reproduced?

The data presented are likely reproducible based on the methodologies described.

Are the experiments adequately replicated and statistical analyses adequate?
Yes, the experiments are adequately replicated, and the statistical analyses are appropriate.

Minor comments on the article, such as specific experimental issues that are easily addressable?

Yes, there are several minor points to consider. The iCre75 line initiates expression at postnatal day 7 (P7), while outer segment formation begins at P9-P10. This raises the question of whether the outer segments observed in P15 mice result from incomplete Cre excision. This could be further clarified. Additionally, the Stx3 staining appears different between control and experimental groups at 12 days post-injection (dpi). A different representative image might enhance clarity.
Furthermore, the authors describe phalloidin staining in control retinas as being intense in the inner segment region, extending into both the nuclear and inner segment layers. Could this be from microvilli? The authors may want to specify that this intense staining could be attributed to the microvilli of Müller glia. The authors could also consider adding PNA staining along with the WGA to show the PNA does not mislocalize as WGA does.

Are prior studies referenced appropriately?

Yes, prior studies are cited appropriately throughout the manuscript. Are the text and figures clear and accurate?
Yes, both the text and figures are presented clearly and accurately.

Suggestions that would help the authors improve the presentation of their data and conclusions?
None

Significance

Provide contextual information to readers (editors and researchers) about the novelty of the study, its value for the field, and the communities that might be interested.

© 2025. Published by The Company of Biologists under the terms of the Creative Commons Attribution License (https://creativecommons.org/licenses/by/4.0/).

This study presents novel findings, including the absence of nascent discs, the presence of extracellular vesicles containing rhodopsin, abnormal Golgi structure, and actin cytoskeleton irregularities across two INPP5E knockout models. These results contribute insights into the cellular mechanisms underlying photoreceptor function loss in INPP5E KO models.

General assessment: provide a summary of the strengths and limitations of the study. What are the strongest and most important aspects? What aspects of the study should be improved or could be developed?

Strengths: This study elucidates a potential mechanism for the observed loss of photoresponse in INPP5E-deficient retinas, demonstrating failures in nascent disc development alongside Golgi and actin cytoskeletal defects.

Limitations: While the study offers valuable insights, the novelty is somewhat limited and evidence for Prom1 mislocalization is not convincing.

Advance: compare the study to the closest related results in the literature or highlight results reported for the first time to your knowledge; does the study extend the knowledge in the field, and in which way? Describe the nature of the advance and the resulting insights (for example: conceptual, technical, clinical, mechanistic, functional, etc.).

Prior studies, such as those by Jacoby et al. (2009) and Sharif et al. (2021), have demonstrated that INPP5E knockout leads to photoreceptor degeneration and impaired outer segment formation. This manuscript builds upon these findings by exploring the role of INPP5E in later stages of outer segment formation and maintenance. The authors provide novel insights into the lack of nascent discs, the presence of rhodopsin-containing extracellular vesicles, and abnormalities in Golgi structures and the actin cytoskeleton in two distinct INPP5E knockout models. However, the study's contribution is somewhat tempered by overlapping results previously reported in the literature. For example, Jacoby et al. (2009) already show that KO of INPP5E in adult mice causes photoreceptor loss and Sharif et al. (2021) shows early retinal KO of INPP5E causes failure of the photoreceptor outer segments to form and presents a model for mechanism based on intraflagellar transport disruption and failure of axoneme formation.

Please describe the type of audience ("specialized," "broad," "basic research," "translational/clinical," etc.) that will be interested or influenced by this research; how will this research be used by others; will it be of interest beyond the specific field?

This manuscript primarily targets a specialized audience engaged in photoreceptor research; however, it will likely also attract interest from the ciliopathy research community due to the implications of INPP5E in ciliary function and mutations in INPP5E being linked to Joubert syndrome.

Please define your field of expertise with a few keywords to help the authors contextualize your point of view. Indicate if there are any parts of the paper that you do not have sufficient expertise to evaluate.

I study photoreceptor cilia.

Author response to reviewers' comments

If you wish to submit a preliminary revision with a revision plan, please use our "Revision Plan" template. ***It is important to use the appropriate template to clearly inform the editors of your intentions.]***

1. General Statements [optional]

© 2025. Published by The Company of Biologists under the terms of the Creative Commons Attribution License (https://creativecommons.org/licenses/by/4.0/).

On behalf of all of the authors, we would like to thank the editorial staff at Review Commons for their handling of our manuscript and also give a big thanks to the reviewers who carefully evaluated the work. We appreciate the comments as the corrections and additions we made in response to the critiques strengthen the manuscript. We have added additional quantification and images as requested by the reviewers and have made numerous edits to the text to clarify and correct issues raised by the reviewers. Detailed point-by-point response to the critiques are presented below.

2. Point-by-point description of the revisions

Reviewer #1 (Evidence, reproducibility and clarity (Required)):

This manuscript provides a partial phenotypic description of knockouts of the Inpp5e gene in the mouse retina at different developmental timepoints: one during the initial expression of rhodopsin in rods and emergence of outer segments (ICre75), and the other after full development of many rod outer segments and 12 days after administration of a knockout-inducing drug (tamoxifen). As far as it goes, the phenotypic characterization seems to be technically sound, although it is carried out at a limited number of time points. The question arises, since this gene has been inactivated previously in mouse retina, "what valuable new information is provided by the present work?" One item is the conclusion that this protein is not only essential for early development of outer segments (already known), but also for renewal of outer segments after rod maturation (new).

1. It is somewhat surprising that there is a detectable b-wave as late as P30 in the iCre75 retinas, suggesting that at least some semblance of outer segments may have developed and survived up to this time point without INPP5E protein. However, that conclusion is rendered somewhat ambiguous by the uncertainty of the lifetime of whatever pool of INPP5E was already present prior to gene inactivation in the later stages of rod development. This caveat is underscored by the observation of a few OS with newly formed discs in the CreERT2 mice 12 days after tamoxifen administration, possibly due to a small remaining pool of functional INPP5E at this time point. This is a point the authors may want to discuss.

It is true that a detectable b-wave is present at P30 in the iCre75 retinas. However, it is significantly reduced as compared to controls. As suggested by the reviewer, this b- wave could result from a failure of the Cre to completely delete all alleles of Inpp5e, from residual Inpp5e activity remaining from the gene prior to deletion or lingering outer segments that are missing Inpp5e but have not fully degenerated. We attempted to directly address this question by immunostaining retinal sections with commercially available Inpp5e antibodies as well as the previously published antibody used by the Baehr laboratory (Sharif et al., 2021).Unfortunately, we were unsuccessful in obtaining signal that was different from secondary antibody-only controls suggesting that it was non-specific. Examination of TEMs at P15 showed that most outer segments had abnormal morphology suggesting that the majority of the rods are missing Inpp5e and that the most explanation is that the signal is due to outer segments that have not fully degenerated. Text now says:

“Scotopic a and b waves were nearly absent at P30, although a residual signal was observed that could be driven by cells where the Cre was not effective or in cells where the outer segments have not yet fully degenerated.”

2. Perhaps the most interesting new observation is the large accumulation of extracellular vesicles in the CreERT2 model, a phenomenon seen previously in retinas of mice with defects in ciliary function or structure. Because these were not observed in the previously published model (although intracellular vesicles were observed) it would be especially interesting to know if these are also formed in the ICre75 mice, but no data are provided to answer that question.

We observe extracellular vesicles in both models. Our electron microscopy analysis of $Inpp5e^{fl/fl}$/iCre75 retinal sections reveals significant EV accumulation, similar to that seen in

© 2025. Published by The Company of Biologists under the terms of the Creative Commons Attribution License (https://creativecommons.org/licenses/by/4.0/).

the Inpp5e$^{fl/fl}$/Pde6g$^{CreERT2/+}$ model. Representative electron micrographs demonstrating EV accumulation in both models are now incorporated into Figure 2.

3. It would not be surprising if these vesicles contain rhodopsin, given its high abundance and the fact that trafficking to OS is largely blocked. However, the immuno- EM data presented are not particularly convincing. There are some black dots on the vesicles, but there are lots of black dots, some colored and some not. At the least, the Methods should describe what criteria were used to distinguish pixels corresponding to antibody staining from others of similar appearance.

We have reselected images to better show visualize the gold labeling, which is densely observed in the vesicles.

4. The immunofluorescence evidence for disruption of Golgi is not particularly strong. A single image each is shown for control and knockout cells, and a single antigen was imaged and the images have low signal-to-noise. Despite the assignment of 4 asterisks to the difference between control and experimental measurements in Fig. 4C, the difference in means is small and the noise is high, without, apparently even taking into account the intrinsic noise in the signal. If such data are to be taken seriously and conclusions drawn from them, details of the image analysis and statistical treatment, which are missing, should be provided in the Methods.

5.

While the changes we detect are subtle, they are consistently observed across multiple samples and also are observed by TEM. We have added detailed methods describing our image acquisition and analysis protocols to ensure transparency in how these measurements were obtained.

6. Finally, the death of cells is not well documented. In the earlier paper such death was obvious, but here there is only documentation of the loss of proper OS structure and some distortion of internal membranes in the IS. It seems likely that loss of OS and rhodopsin mislocalization ultimately would lead to extensive, if not complete, loss of rod nuclei but no evidence is provided that bears on that hypothesis. This weakness is somewhat disappointing, considering the technical feasibility of such experiments, once the animals are in hand. Although the manuscript refers to "rapid degeneration" it is not obvious that much cell death has occurred at the time points sampled.

Initial characterization documented significant photoreceptor loss at later timepoints. Our interest is in the events that precede photoreceptor loss and so we strategically focused our electron microscopy and immunofluorescence analyses on early timepoints. This approach was chosen to distinguish primary defects from secondary consequences of degeneration, allowing us to better understand the molecular mechanisms underlying Inpp5e function. However, early in the analysis we analyzed Pde6g-CreERT2 mice 30 days after tamoxifen injection. These animals showed significant thinning of the outer nuclear layer, near-complete absence of outer segments, and disrupted retinal architecture, confirming that the early structural changes we characterized ultimately lead to photoreceptor degeneration. To address the reviewer’s concern, this data is now presented as Sup. Fig 1.

CROSS-REFEREE COMMENTS
I think it should not take too long for the authors to address the concerns raised by all three reviewers, who seem to agree that the manuscript is of value but has some weaknesses.

Reviewer #2 (Evidence, reproducibility and clarity (Required)):

This study examined the role of INPP5E in transciliary trafficking in retinal photoreceptors. Human patients with INPP5E pathogenic variants develop retinal degeneration. While the role of INPP5E in ciliary trafficking have been reported before, the current study utilized tissue specific and inducible promoter constructs to inactivate INPP5E at both the onset of photoreceptor outer

© 2025. Published by The Company of Biologists under the terms of the Creative Commons Attribution License (https://creativecommons.org/licenses/by/4.0/).

segment morphogenesis and in mature photoreceptors, thus providing a fuller view of the role of INPP5E in retinal photoreceptors. The study finds a slew of cell biological defects including rhodopsin mis-localization to the inner segments, malformation of disc membranes and disruption of actin filament networks that are thought to help extend nascent discs at the base of outer segments.

Major comment: The study is well designed and carried out. The data are of high quality and support their conclusions. It provides new insights to cilia biology and function of INPP5E.

Thank you!

1. Minor comment: Page 3, 5th line from bottom: PI(4)P2 should be PI(4)P.
   Thank you, this has been corrected to PI(4)P.

Reviewer #2 (Significance (Required)):

The study contributes new knowledge to the pathophysiological process related to INPP5E mutations, although it does not examine these issues at the molecular detail.

The manuscript is of interest to researchers who are generally interested in retinal degeneration, cilia biology and ciliopathy.

Reviewer #3 (Evidence, reproducibility and clarity (Required)):

Summary
This manuscript investigates the role of inositol polyphosphate-5-phosphatase E (INPP5E) in the development and maintenance of photoreceptors. Utilizing two distinct animal models, the authors examine the effects of INPP5E knockout in rod photoreceptors during two critical developmental stages: early retinal development, when photoreceptors are post-mitotic, and post-developmental stages. The findings reveal that the absence of INPP5E results in: 1) diminished rod photoresponse, 2) failure of nascent photoreceptor disc formation, 3) mislocalization of rhodopsin with subsequent accumulation of rhodopsin-containing vesicles near the inner segments, 4) structural disruption of the Golgi apparatus, and 5) alterations to the actin cytoskeleton, as evidenced by F-actin staining. The authors conclude that the observed reduction in photoresponse is likely attributed to the failure of photoreceptors to develop new outer segments and that INPP5E plays a critical role in protein trafficking.

Major comments on the article

1. I recommend that the authors include Western blot analysis and/or immunostaining for INPP5E in the knockout models to validate the effectiveness of the genetic manipulations.

Western blot analysis would need to be done on whole retina, which is composed of multiple other cell types besides the rods where we are deleting Inpp5e. As we do not know anything about the relative amounts of Inpp5e in the various cell types, we would not be able to interpret the results of the western blot. Immunofluorescence would allow us to observe Inpp5e levels on a cell-by-cell basis. However, we attempted retinal staining with commercially available antibodies as well as the one published previously (Sharif et al., 2021) with no signal beyond what is observed in secondary-only controls, indicating that it is background.

Despite these technical limitations, the severe phenotypes observed in both our iCre75 and CreERT2 models provide strong indirect evidence for the effectiveness of Inpp5e knockout. The rapid loss of outer segment structure, mislocalization of rhodopsin, and eventual photoreceptor cell death are consistent with successful deletion of Inpp5e.

2. Additionally, the immunohistochemical (IHC) images demonstrating Prom1 mislocalization do not convincingly support the claim. To strengthen this aspect, it would be beneficial to provide multiple images from various retinal regions. Additionally, the study by Sharif et al. (2021)

© 2025. Published by The Company of Biologists under the terms of the Creative Commons Attribution License (https://creativecommons.org/licenses/by/4.0/).

indicates that Prom1 does not mislocalize in the INPP5E knockout using the Six3Cre line; while the authors acknowledge this discrepancy, they do not offer a potential explanation.

We have quantified Prom1 fluorescence intensity in the inner segments of control and mutant retinas. The new quantification shows a significant increase in Prom1 intensity in mutant inner segments compared to controls. This quantitative data is now presented in Figure 5 alongside representative images, with individual data points shown to demonstrate the consistency of this finding across multiple samples.

3. Another point to consider is that there could be additional markers used to confirm Golgi dysfunction as well as disruption of the F-actin network. Particularly, additional evidence for F-actin network disruption should be considered given that the Wasf3 staining looked similar between the experimental and control mice.

Our study utilized phalloidin for F-actin and giantin for the Golgi apparatus, both well-established markers in the field. While additional markers could offer complementary information, we believe our current data provide valuable insights into the structural changes occurring in our model.

Regarding Wasf3 staining, it's important to note that Wasf3 reflects actin nucleation initiators, while phalloidin directly labels F-actin filaments. The similarities in Wasf3 staining between experimental and control mice, contrasted with the changes observed in phalloidin staining, suggest that while the actin nucleation machinery remains largely intact, the overall F-actin network is disrupted. This distinction highlights the complex regulation of actin dynamics in our model, which we discuss further in the manuscript.

4. The claims presented are largely supported by the data; however, the assertion that INPP5E is necessary for protein trafficking could benefit from further clarification. It remains unclear whether this necessity stems from a direct interaction with specific proteins or is a consequence of previously established impairments in axoneme extension and abnormal localization of intraflagellar transport (IFT) particles observed in INPP5E knockout models.

We have removed the statement about involvement in trafficking from the abstract and the last paragraph of the introduction. Title has been changed to: *Inpp5e Is Critical for Photoreceptor Outer Segment Maintenance*

Do you have constructive further-reaching suggestions that could significantly improve the study but would open new lines of investigation? Please label them as "OPTIONAL".

Are the suggested experiments realistic in terms of time and resources? It would help if you could add an estimated time investment for substantial experiments.

Yes, the proposed experiments appear realistic regarding time and resource requirements.
Are the data and the methods presented in such a way that they can be reproduced? The data presented are likely reproducible based on the methodologies described. Are the experiments adequately replicated and statistical analyses adequate?
Yes, the experiments are adequately replicated, and the statistical analyses are appropriate.

Minor comments on the article, such as specific experimental issues that are easily addressable?

1. Yes, there are several minor points to consider. The iCre75 line initiates expression at postnatal day 7 (P7), while outer segment formation begins at P9-P10. This raises the question of whether the outer segments observed in P15 mice result from incomplete Cre excision. This could be further clarified.

© 2025. Published by The Company of Biologists under the terms of the Creative Commons Attribution License (https://creativecommons.org/licenses/by/4.0/).

This is an important question about the timing of iCre75 expression relative to outer segment development. While iCre75 expression begins at P7 and outer segment formation initiates at P9-P10, several factors support the validity of our P15 observations. Previous characterization of the iCre75 line has demonstrated efficient and rapid recombination in rod photoreceptors, with the two-day window between Cre expression and outer segment initiation allowing sufficient time for significant gene deletion. The severity of outer segment defects we observe at P15 indicates substantial Inpp5e loss, as incomplete excision would likely result in a milder phenotype. Additionally, our observations in the iCre75 model align well with those from our inducible CreERT2 model, and the progressive worsening of the phenotype from P15 onwards is consistent with effective early Cre-mediated recombination. To address this concern, we added the following text:

“Outer segment elongation starts at about P9/P10 (LaVail, 1973; Obata and Usukura, 1992; Pearring et al., 2013), thus it is expected that pathology would start during the elongation phase due to the time required for the gene to be deleted and the gene products decay.”

2. Additionally, the Stx3 staining appears different between control and experimental groups at 12 days post-injection (dpi). A different representative image might enhance clarity.

The interpretation of Stx3 localization in the experimental group is complicated by the presence of degenerating outer segments that have collapsed into the inner segment layer. This structural disorganization makes it challenging to definitively distinguish Stx3 localization patterns. However, in regions where we can clearly identify intact outer segments, we do not observe significant mislocalization of Stx3, unlike what has been reported in some other models of retinal degeneration.

3. Furthermore, the authors describe phalloidin staining in control retinas as being intense in the inner segment region, extending into both the nuclear and inner segment layers. Could this be from microvilli? The authors may want to specify that this intense staining could be attributed to the microvilli of Müller glia.

We are not certain that we understand the reviewer’s comment. If the reviewer is suggesting that all of the phalloidin label that we observe is from Muller glia, we can say that staining with glutamine synthetase antibodies did not reveal any differences in Müller glial morphology or distribution between control and knockout retinas suggesting that the loss of phalloidin label in the inner segment is not due to a loss of glia cells. If the reviewer is suggesting that the remaining label in the knockout is due to actin in the Muller glia, we would agree and have added a statement to text

“This is the location of Muller glia, which have an extensive actin cytoskeleton (Tworig and Feller, 2021) and could be contributing to phalloidin signal in this region.”

4. The authors could also consider adding PNA staining along with the WGA to show the PNA does not mislocalize as WGA does.

PNA specifically marks cone photoreceptor outer segments and synaptic terminals (Blanks and Johnson, 1984; Hageman and Johnson, 1986) Our existing cone opsin immunostaining shows normal localization patterns, which is expected given our rod-specific INPP5E knockout model. Thus, PNA is not expected to provide any additional information.

Are prior studies referenced appropriately?
Yes, prior studies are cited appropriately throughout the manuscript.

Are the text and figures clear and accurate?

© 2025. Published by The Company of Biologists under the terms of the Creative Commons Attribution License (https://creativecommons.org/licenses/by/4.0/).

Yes, both the text and figures are presented clearly and accurately.

Suggestions that would help the authors improve the presentation of their data and conclusions? None

Reviewer #3 (Significance (Required)):

Provide contextual information to readers (editors and researchers) about the novelty of the study, its value for the field, and the communities that might be interested.

This study presents novel findings, including the absence of nascent discs, the presence of extracellular vesicles containing rhodopsin, abnormal Golgi structure, and actin cytoskeleton irregularities across two INPP5E knockout models. These results contribute insights into the cellular mechanisms underlying photoreceptor function loss in INPP5E KO models.

General assessment: provide a summary of the strengths and limitations of the study. What are the strongest and most important aspects? What aspects of the study should be improved or could be developed?

Strengths: This study elucidates a potential mechanism for the observed loss of photoresponse in INPP5E-deficient retinas, demonstrating failures in nascent disc development alongside Golgi and actin cytoskeletal defects.

Limitations: While the study offers valuable insights, the novelty is somewhat limited and evidence for Prom1 mislocalization is not convincing.

Advance: compare the study to the closest related results in the literature or highlight results reported for the first time to your knowledge; does the study extend the knowledge in the field, and in which way? Describe the nature of the advance and the resulting insights (for example: conceptual, technical, clinical, mechanistic, functional, etc.).

Prior studies, such as those by Jacoby et al. (2009) and Sharif et al. (2021), have demonstrated that INPP5E knockout leads to photoreceptor degeneration and impaired outer segment formation. This manuscript builds upon these findings by exploring the role of INPP5E in later stages of outer segment formation and maintenance. The authors provide novel insights into the lack of nascent discs, the presence of rhodopsin- containing extracellular vesicles, and abnormalities in Golgi structures and the actin cytoskeleton in two distinct INPP5E knockout models. However, the study's contribution is somewhat tempered by overlapping results previously reported in the literature. For example, Jacoby et al. (2009) already show that KO of INPP5E in adult mice causes photoreceptor loss and Sharif et al. (2021) shows early retinal KO of INPP5E causes failure of the photoreceptor outer segments to form and presents a model for mechanism based on intraflagellar transport disruption and failure of axoneme formation.

We have discussed how our results confirm and extend the work of Jacoby et al. (2009) and Sharif et al. (2021). However, the reviewer’s statement “Jacoby et al. (2009) already show that KO of INPP5E in adult mice causes photoreceptor loss” is over stating what is in this work. Jacoby et al. (2009) show one low magnification histological image of a retina 5 months after tamoxifen treatment.

Please describe the type of audience ("specialized," "broad," "basic research," "translational/clinical," etc.) that will be interested or influenced by this research; how will this research be used by others; will it be of interest beyond the specific field?

This manuscript primarily targets a specialized audience engaged in photoreceptor research; however, it will likely also attract interest from the ciliopathy research community due to the implications of INPP5E in ciliary function and mutations in INPP5E being linked to Joubert syndrome.

Please define your field of expertise with a few keywords to help the authors contextualize your

© 2025. Published by The Company of Biologists under the terms of the Creative Commons Attribution License (https://creativecommons.org/licenses/by/4.0/).

point of view. Indicate if there are any parts of the paper that you do not have sufficient expertise to evaluate.

I study photoreceptor cilia.

Blanks, J.C., and L.V. Johnson. 1984. Specific binding of peanut lectin to a class of retinal photoreceptor cells. A species comparison. *Invest Ophthalmol Vis Sci*. 25:546-557. PMID: 6715128.

Hageman, G.S., and L.V. Johnson. 1986. Biochemical characterization of the major peanut-agglutinin-binding glycoproteins in vertebrate retinae. *J Comp Neurol*. 249:499-510, 482-493. PMID: 3745505. 10.1002/cne.902490406.

LaVail, M.M. 1973. Kinetics of rod outer segment renewal in the developing mouse retina. *J Cell Biol*. 58:650-661. PMID: 4747920. 10.1083/jcb.58.3.650. PMC2109077

Obata, S., and J. Usukura. 1992. Morphogenesis of the photoreceptor outer segment during postnatal development in the mouse (BALB/c) retina. *Cell Tissue Res*. 269:39-48. PMID: 1423483. 10.1007/BF00384724.

Pearring, J.N., R.Y. Salinas, S.A. Baker, and V.Y. Arshavsky. 2013. Protein sorting, targeting and trafficking in photoreceptor cells. *Prog Retin Eye Res*. 36:24-51. PMID: 23562855. 10.1016/j.preteyeres.2013.03.002. PMC3759535

Sharif, A.S., C.D. Gerstner, M.A. Cady, V.Y. Arshavsky, C. Mitchell, G. Ying, J.M. Frederick, and W. Baehr. 2021. Deletion of the phosphatase INPP5E in the murine retina impairs photoreceptor axoneme formation and prevents disc morphogenesis. *J Biol Chem*. 296:100529. PMID: 33711342. 10.1016/j.jbc.2021.100529. PMC8047226

Original submission

First decision letter

MS ID#: JOCES/2024/263814

MS TITLE: Inpp5e Is Critical for Photoreceptor Outer Segment Maintenance

AUTHORS: Mohona Gupta, Taylor Lewis, Michael W. Stuck, William J. Spencer, Natalia V. Klementieva, Vadim Arshavsky and Gregory J. Pazour
ARTICLE TYPE: Research Article

Dear Dr Pazour,

Thank you for sending your manuscript to Journal of Cell Science through Review Commons.

Based on your thorough revisions to the reviewers questions from Review Commons, I am happy to tell you that your manuscript has been accepted for publication in Journal of Cell Science, pending standard publication integrity checks. It was accepted on 07 Jan 2025. Where referee reports on this version are available, they are appended below.

© 2025. Published by The Company of Biologists under the terms of the Creative Commons Attribution License (https://creativecommons.org/licenses/by/4.0/).
